# Machine learning based, subject-specific, gender and race independent, non-invasive estimation of the arterial blood pressure

**DOI:** 10.1038/s44325-025-00075-5

**Published:** 2025-08-01

**Authors:** Rahul Kumar Sevakula, Patrícia J. Bota, Mohamad B. Kassab, Sandeep Chandra Bollepalli, Geerthy Thambiraj, Richard Boyer, Eric M. Isselbacher, Antonis A. Armoundas

**Affiliations:** 1 Cardiovascular Research Center, Massachusetts General Hospital, Boston, MA USA; 2 Anesthesia Department, Massachusetts General Hospital, Boston, MA USA; 3 Healthcare Transformation Lab, Massachusetts General Hospital, Boston, MA USA; 4 Institute for Medical Engineering and Science, Massachusetts Institute of Technology, Cambridge, MA USA

**Keywords:** Hypertension, Health care, Electrocardiography - EKG, Hypertension

## Abstract

Software-based blood pressure (BP) measurement offers non-invasive, continuous, real-time monitoring compared to traditional methods. This study explores a non-invasive machine learning approach to estimate arterial BP from ECG and SpO_2_ signals, using intra-arterial catheter BP readings as ground truth. A random forest (RF) algorithm was trained on a large dataset (~30 M beats, ~400 patient days), using extracted signal morphological features and patient characteristics. The RF model achieved low mean absolute error (MAE) for systolic/diastolic BP (4.29 ± 5.00 mmHg/2.38 ± 3.25 mmHg), independent of gender and race. Personalized models further improved performance (MAE: 3.51 ± 4.24 mmHg/1.85 ± 2.60 mmHg). We assessed different ECG lead combinations for estimating BP and observed that two limb leads, or a precordial lead were sufficient for an estimation below 5 mmHg MAE. These findings highlight the potential of real-time, personalized BP monitoring for early detection of hypertension, enhancing healthcare accessibility through non-invasive, continuous monitoring.

## Introduction

Ambulatory blood pressure (BP) monitoring has numerous advantages over in-office BP measurements in diagnosing and managing hypertension^1,2^, such as the feasibility of measuring BP during sleep^3^, the ability to evaluate the effect of drug regimens^4^, and the mitigation of systematic errors related to white coat hypertension^5,6^ and terminal digit bias by providers^7^. Population^8^ and meta-analysis^9^ studies have suggested that 24 h ambulatory BP monitoring adds prognostic utility compared with office BP readings alone, and that night-time ambulatory BP is superior to daytime ambulatory BP for determining cardiovascular mortality^8–10^.

The majority of the ambulatory BP monitors today are automated oscillometric devices, which measure brachial BP and require a cuff be placed on the upper arm. Discomfort due to the cuff, particularly with repeated measurements, is a major concern for patients^11^, and limits the feasibility of night-time measurements. Furthermore, it has been found that automated oscillometric devices exhibit inherent error during BP measurement, and are more variable than the invasive ABP monitors^12,13^, which are the gold-standard for arterial pressure monitoring^14^. A few ambulatory BP monitors have recently introduced calibration techniques and software methods to estimate the central ABP^15^. Other notable examples of portable, periodic, non-invasive BP monitoring are ultrasonic device-based methods^16^ and software-based BP estimation methods^17–19^. Ultrasonic devices have yet to arrive for mainstream use in continuous monitoring. Fortunately, there is much evidence in the literature^20–24^ to indicate the feasibility of estimating BP from oxygen saturation (SpO_2_) and electrocardiographic (ECG) signals using software-based methods.

Software-based BP estimation methods can be divided into different categories based on: (1) which physiological signals are used as input, and (2) the gold-standard BP (i.e., branchial cuff BP vs. arterial BP). The inputs and the application use-case are strongly interdependent. The dependency of BP on the PPG signal is so well established^25^ that the majority of the software-based BP estimation methods have used PPG as one of the inputs for BP estimation. In fact, several methods^18–20,24–36^ (see Supplementary Table 1) have based their BP estimation method on PPG signals acquired from one or multiple sources alone. The pulse transit time (PTT) and pulse arrival time (PAT) are the two most popular and well-studied measures to indicate the speed of BP waves, as an indicator of BP; PTT refers to the time duration that the pulse takes to travel between two arterial sites, and PAT refers to the time duration between the R-wave of ECG, to the foot of PPG^37^. PTT can be found either using a single source PPG and the ECG signal, or by using PPG signals from two sensors at different locations^38^. PAT, on the other hand, requires both PPG as well as ECG signals. This brings us to methods^17,23,39–41^ that use not only PPG, but ECG as well. Of them, the majority^23,39,41,42^ have used cuff-based BP measurements as the gold-standard. As noted earlier, the true gold-standard for recording ABP on a beat-to-beat basis uses an intra-arterial catheter; however, this requires an invasive procedure that causes discomfort and poses a risk of infection or arterial injury, so it is performed only in critically ill or surgical patients. Having non-invasive, software-based methods to estimate the ABP in real-time with reasonably low error is therefore a very attractive proposition--even more so if one considers the catheter-based ABP as the gold-standard. Few software based methods have tried doing the same with PPG alone^18,19,25,36^ or with the PPG and ECG together^17^. It should be noted that all these studies employed physiological signal repositories pertaining to patients admitted to the ICU. Notable repositories include Medical Information Mart for Intensive Care III (MIMIC III)^42^, UCI Cuff-Less Blood Pressure Estimation Data Set^17^, and the University of Queensland Vital Signs Dataset^43^.

This study presents a machine learning (ML) method, which has been trained over large amounts of telemetry data containing ECG, SpO_2_, ABP signals from patients admitted to the ICU, to estimate the systolic/diastolic BP. To our knowledge, this is among the largest datasets that have ever been trained/validated for BP estimation^17,36,43,44^. Compared to earlier studies^25^, our method is able to estimate the systolic/diastolic values of ABP with the lowest mean error, and can perform the BP estimation on a beat-to-beat basis. The low error can be attributed to our ML approach, the large volume of data available for training, and the fact that we use information from both SpO_2_ and ECG signals, as well as the feature selection. Furthermore, the present study also analyzes the number/combinations of ECG leads required for optimal performance, and the feasibility and impact of developing subject customized BP estimation models.

## Results

### Model hyperparameter tuning

We first sought to determine the optimal number of trees in RF, with respect to robustness and efficiency in estimating the systolic and diastolic BP (please see Supplementary Table 2), that resulted in choosing 400 tree RF models.

### Algorithm performance evaluation across varying sequence lengths

A widely accepted SP10 Protocol by the Association for the Advancement of Medical Instrumentation (AAMI) proposes that a BP monitoring device’s error tolerance should be below 5 mmHg^45,46^. To that effect, we have adopted to report the performance of the present BP estimation analysis in terms of mean absolute error (MAE), as many other studies before^17,25,40^.

**Fig. 1:**
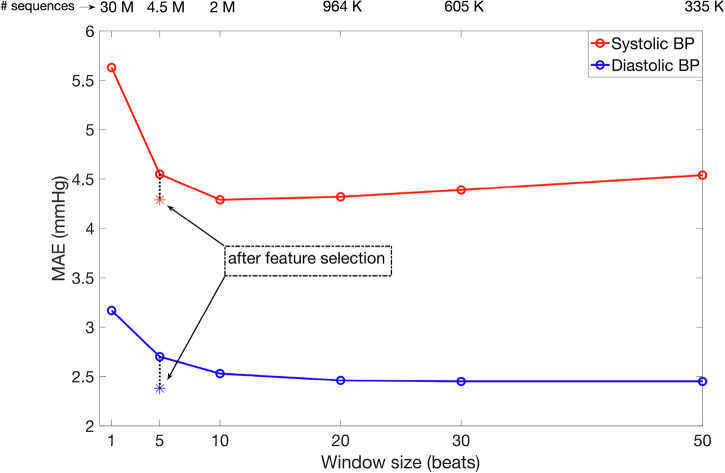
Blood pressure estimation performance across different window-lengths. The plot shows the mean absolute error (MAE) achieved during systolic/diastolic blood pressure (BP) estimation across different lengths of windows/sequences. There is significant improvement in performance for length > 1 beat, after which the differences are minor. Feature selection further improves the performance. The number of sequences participating in each experiment is mentioned at the top of the figure.

Next, we evaluated the performance of the method in estimating the systolic and diastolic BP across different window lengths, employing RF models consisting of 400 trees. Since all sequences were non-overlapping, longer sequences resulted in fewer sequences. The results are shown in Fig. 1, where we can observe that the MAE in estimating systolic/diastolic BP is below 5 mmHg for {5, 10, 20, 30, 50} beat sequences; furthermore, the MAE difference across these window lengths has been very small. Of all window lengths, we chose the 5-beat one, because: (1) the BP estimation error was reasonably low, and (2) the BP estimation with 5-beat sequence would reflect a more instantaneous estimate of the BP than windows of length {10,20,30,50}.

### Model feature selection

Next, we performed feature selection, and observed that the subject’s race, gender, fractal dimension feature from ECG, and coefficients from the auto-regressive model fitted over SpO_2_ are not important features (rank of each feature is provided in Supplementary Table 3, Supplementary Figs. 1, 2), whereas the subject’s age, BMI, T-wave amplitude, heart rate, and ECG signal mobility, are important features. We have noticed that by eliminating the less important features, the MAE of the systolic/diastolic BP estimation improved to (4.29 ± 5.00)/(2.38 ± 3.25) mmHg, respectively, leading to the conclusion that the initial set of features could result in overfitting, and thus to reduction of the model performance.

To examine the importance of gender and race in BP estimation, we have separately investigated whether the gender and race features affected the BP estimation. Addition of gender resulted in MAE of systolic/diastolic BP estimates of (4.29 ± 5.00)/(2.39 ± 3.25) mmHg, respectively, while addition of race resulted in MAE of (4.52 ± 5.25)/(2.67 ± 3.61) mmHg, respectively. We observe that gender and race do not improve the estimation performance of the algorithm, while age is a predominant factor. Importantly, this shows that the BP estimation algorithm does not need calibration for the subject’s gender and race.

**Fig. 2:**
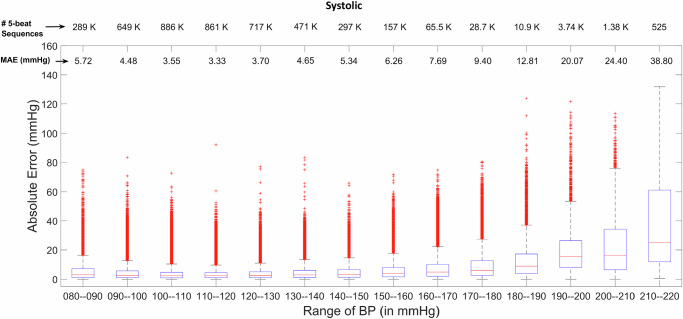
Mean absolute error of systolic and diastolic BP across different BP values. Box-plots providing performance analysis of the proposed method in estimating systolic blood pressure (BP) across different BP ranges. The number of employed sequences pertaining to each BP range is mentioned at the top of the two figures. Box plots indicate the distribution of absolute errors in arterial BP estimation across very broad BP values. The blue box spans the interquartile range (IQR, 75–25%), covering the median (50%, red line) of the data from the first quartile (Q1, 25th percentile) to the third quartile (Q3, 75th percentile). The black caps (upper and lower horizontal lines extend to +− 1.5 times IQR) indicate the furthest data points that are still within the expected range before classifying values as outliers. Red markers indicate outliers, representing extreme errors.

**Fig. 3:**
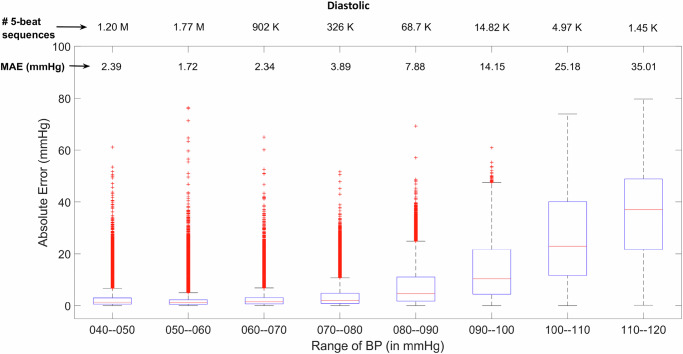
Mean absolute error of systolic and diastolic BP across different BP values. Box-plots providing performance analysis of the proposed method in estimating diastolic BP, across different BP ranges. The number of employed sequences pertaining to each BP range are mentioned at the top of the two figures. Box plots indicate the distribution of absolute errors in arterial BP estimation across very broad BP values. The blue box spans the interquartile range (IQR, 75–25%), covering the median (50%, red line) of the data from the first quartile (Q1, 25th percentile) to the third quartile (Q3, 75th percentile). The black caps (upper and lower horizontal lines extend to +− 1.5 times IQR) indicate the furthest data points that are still within the expected range before classifying values as outliers. Red markers indicate outliers, representing extreme errors.

Figures 2, 3 present box-plots of the systolic and diastolic BP estimation absolute error, across a wide range of gold-standard ABP values. These results suggest that the model performs well in estimating the systolic/diastolic BP between (80–160) mmHg/(40–80) mmHg, respectively; however, the main reason for performing less well outside of these ranges was the limited availability of data (seen at the top of each of these figures).

### Performance evaluation across demographic features

Supplementary Figs. 3–8 display the general model’s absolute error for systolic and diastolic BP across age (Supplementary Figs. 3 and 4, respectively), gender (Supplementary Figs. 5, 6, respectively) and race (Supplementary Figs. 7 and 8, respectively) groups. The results indicate that across all age, gender, and race groups, the majority of the estimated MAEs fall within a narrow and consistent range, demonstrating stable model performance across these demographic categories. Employing the LMM we observed that the general model performed equally well across different ages (Systolic: *p* = 0.79, Diastolic: *p* = 0.73), genders (Systolic: *p* = 0.27, Diastolic: *p* = 0.09), and self-declared races (Systolic: *p* = 0.22, Diastolic: *p* = 0.29).

### Beat-by-beat BP estimation

To assess the accuracy of a more instantaneous BP estimation approach, we sought to advance the 5-beat window ahead by only one beat at a time, and continually update the BP estimate, over every new 5-beat window. Naturally, the windows in such a scenario, would be overlapping, where a window may have overlapping beats with up to 4 prior or subsequent windows; therefore, during the five-fold cross-validation, we first decided the test data and then, from the remaining data that was available for train, we filtered out all windows that could have any overlap with test data by rejecting four overlapping sequences prior to and following the test window. Following this approach, even though we had ~18 M 5-beat overlapping windows available, only ~2.8 M windows were used for train and ~3.7 M windows for test. Following this approach, the MAE during systolic/diastolic BP estimation was (4.37 ± 5.14)/(2.42 ± 3.31) mmHg, respectively; the counterintuitive minor drop in performance may be attributed to the reduction of the available training data. The analysis henceforth was performed only with 5-beat non-overlapping sequences.

### Algorithm performance evaluation employing a varying number of ECG leads

After estimating BP with SpO_2_ and 4-lead ECG, we next sought to assess how BP estimation performance varies by the number of input ECG leads, and in addition, to identify those ECG lead combinations that are most effective in the performance of the algorithm.

The summary performance results across different lead combinations, with varying number of ECG leads, are presented in Table 1, and the performance results of individual combinations of ECG leads are provided in Supplementary Table 4. The results indicate that: (i) the BP estimation performance declines, albeit mildly, with reduction in number of input ECG leads, (ii) at-least two limb leads or a precordial lead are required to estimate the systolic/diastolic BP within reasonable MAE tolerance of 5 mmHg, and (iii) lead combinations that include the precordial lead always perform better than the combinations where the precordial lead is replaced with a limb lead, thus, indicating the importance of precordial lead in BP estimation.

**Table 1:** BP estimation performance using SpO_2_ and ECG from different numbers of leads

# ECG Leads	Systolic BP estimation MAE (mmHg)	Diastolic BP estimation MAE (mmHg)
4 Leads	4.29	2.38
3 Leads	4.44 ± 0.09	2.45 ± 0.04
2 Leads	4.68 ± 0.12	2.56 ± 0.05
1 Lead	5.21 ± 0.15	2.79 ± 0.06

The mean and standard deviation have been computed by taking the mean and standard deviation across all combinations of the given number of leads. In all cases, BP has been estimated with the 34 selected features.

### Subject specific BP model development and evaluation

We have observed that the performance of our BP estimation method is independent of gender and race, whereas inclusion of the subject’s BMI and age improve the performance, and makes the method tailored to that subject. Additionally, we analyzed the impact on the model’s performance of including and excluding key demographic features such as HR, BMI, and age, identified as top features during the feature selection process (see Supplementary Table 3).

**Fig. 4:**
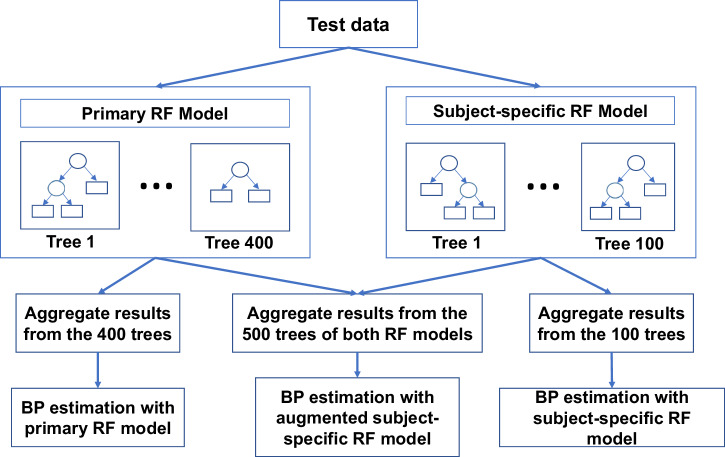
Augmented patient-specific model. This figure describes the augmented patient specific model which takes into account the BP estimates given by both the primary RF model and the corresponding patient specific model of the given patient. The primary RF model is trained across all samples in the training data, and the multiple patient specific RF models (one per patient) are trained over samples of each patient. The augmented patient-specific model ensures that the method is tailored towards the patient and also that it is not subjected to dataset-shift issues, which occur due to lack of samples in a certain BP range.

We, therefore, sought to examine whether it would be feasible in developing a more subject tailored BP estimation, utilizing his/her physiological signals (Fig. 4).

For this analysis, we performed additional steps during the regular five-fold cross-validation procedure. Specifically, we first mapped the train and test data (each 5-beat sequence) to the corresponding 282 patients. Then, during the training phase, we not only trained the primary 400 tree RF model over all training sequences but also trained 282 independent RF models (one per subject) on sequences pertaining to each individual patient; therefore, we coin them as subject-specific models. During the test-phase of the cross-validation, the systolic/diastolic BP was estimated on the test sequences in three different ways:

(i) with the primary RF model having 400 trees alone;
(ii) with the subject-specific models, where test sequences pertaining to each subject are tested upon by the corresponding subject-specific model;
(iii) with the augmented subject-specific model (as explained below).

RF during testing, estimates the BP with each of the decision trees and takes the mean value as the final estimate. With the primary RF model, the estimation is performed with 400 trees, and with the subject-specific model, the same is accomplished with the fixed number of trees it was trained with. In the augmented subject-specific model, we account the BP estimates from trees of both RF models, the primary model, as well as the subject-specific model. To determine the number of trees for the subject-specific RF models, we measured the test performance with each of the three methods, while varying the number of trees of the subject-specific RF models across {50, 100, 200, 300, 500} (this analysis is detailed in Supplementary Table 5), and found 100 trees to be the best choice, with respect to efficiency and robustness. We have found that the subject-specific models achieved a MAE of (3.51 ± 4.24)/(1.85 ± 2.60) mmHg, and the augmented subject-specific model achieved a MAE of (4.10 ± 4.72)/(2.27 ± 3.00) mmHg, respectively, which compared favorably with respect to the primary model that achieved a MAE of (4.29 ± 5.00)/(2.38 ± 3.25) mmHg, respectively (Supplementary Table 6).

The results suggest that the subject-specific models help improve the performance. A concern with subject-specific models is that the amount of data used in training is too short to capture the likely data variability. We have observed that subject-specific models are easily susceptible to dataset-shift issues^47^, i.e., when train data does not contain sequences of a certain BP range, the model would perform poorly when such sequences appear in the test data. In the above studies, we took great care to ensure that the train data contained sequences across the BP range for each subject; accordingly, results have been very positive. Augmenting the subject-specific models can alleviate the dataset-shift concern to a good degree, since the primary RF model is trained on large amounts of data encompassing the entire BP range. On that account, we propose the augmented subject-specific model to be the best performing one among the three.

The augmented subject-specific model’s performance across the 5 folds is presented in Supplementary Figs. 9, 10. One observes that the model performs similarly across the 5 different test-data distributions (linear mixed model, *p*-value > 0.01). Importantly, using the augmented subject-specific model, the correlation between the estimated and the true BP values, across all subjects (Supplementary Figs. 11, 12), shows a high correlation coefficient (>0.9), indicating a strong linear relationship between the model’s predictions and the true values.

Next, we sought to further probe the agreement between the true BP values and the model-estimated BP values by plotting the difference between the true and estimated values against the mean of the true and estimated ABP values (a Bland-Altman plot, Supplementary Figs. 13, 14), across all subjects. One observes that the mean difference (red dashed line) is close to zero (0 mmHg/0.04 mmHg, for systolic/diastolic BP estimation, respectively), indicating a small difference between the model’s estimated and the true values. The limits of the agreement (blue dashed lines, computed as mean difference ± 1.96 standard deviations) suggest that the majority of BP estimations fall within a reasonable error range (<13 mmHg and <7 mmHg, for systolic and diastolic BP estimation, respectively).

Lastly, we probed the augmented subject-specific model’s ability to dynamically track the observed BP amplitude fluctuations. Supplementary Figs. 15, 16 demonstrate an example where the augmented subject-specific model (in green) is able to successfully track BP amplitude changes (in blue). Additionally, a 20-sample windowed cross-correlation has resulted in maximum correlation for a time-lag of 0 samples, indicating the model’s robustness in capturing both gradual and abrupt amplitude changes in BP with no lag. A 20-sample window was used to capture local shifts in BP amplitude without being overshadowed by long-term trends, while also mitigating the effects of non-stationarity by ensuring that correlations are computed within a stable, short-term segment of the signal.

## Discussion

Many studies have suggested that the average ambulatory BP, recorded during 24 h, provides better information to conventional BP measured in a doctor’s office^38,48^. The European Society of Cardiology (ESC) Guidelines recommend the use of the average ambulatory BP, although the ESC has also endorsed the SCORE (Systematic Coronary Risk Evaluation) chart, which is based on office BP measurement^49,50^. The majority of the current 24 h ambulatory BP monitors are cuff-based automated oscillometric devices. The utility of these devices is limited by subject discomfort^11^ and associated loss of sleep^51^, variability/poor accuracy^12^, and low periodicity (every 10–30 min) in obtaining the BP estimates^52^. Such limitations have fostered significant interest in building ultrasonic devices and software-based methods to estimate the ABP on a more frequent/real-time basis.

We hypothesized that RF, applied on an immense and diverse data cohort, would be able to estimate the systolic/diastolic BP, on a beat-to-beat basis, with a high degree of fidelity. This study, performed on data collected in a major metropolitan hospital that sees very diverse demographically patients with very diverse pathophysiological conditions, addressed fundamental issues pertinent to the in- and out-of-hospital patient, such as efficiency (i.e., noise detection, number of required heart beats, overlapping vs. non-overlapping segments of beats, etc.), accuracy and robustness (i.e., feature selection, model development, selection of type of model type that is-general vs. subject-specific, etc.), and ease of use (e.g., the selection of ECG leads). The method was trained by employing the BMI, age, and features from ECG and SpO_2_ signals as inputs, and the intra-arterial systolic/diastolic BP as the gold-standard. Several novel advances have been made from this study: first, to our knowledge, the proposed model was trained on the largest and most demographically diverse data set to date; second, our method performed very well with a MAE of (4.29 ± 5.00 mmHg)/(2.38 ± 3.25 mmHg) for systolic/diastolic BP estimation; third, our method does not need calibration for the gender or race of the subject; fourth, we analyzed the method’s BP estimation ability with SpO_2_ and different number/combinations of ECG leads, and concluded that SpO_2_ in association with at-least two limb leads or a single precordial lead, is enough to obtain BP estimates with a MAE <5 mmHg; fifth, we established the feasibility of making the BP estimation method tailored towards the subject’s age, BMI and physiological signals; sixth, to our knowledge, we use one of the few databases with true ABP, thus minimizing the propagating error resulting from a limb measuring device; and, seventh, an augmented subject-specific model was found to exhibit the lowest MAE compared to the primary RF model, and therefore be most suitable for real-world implementation.

Furthermore, our model’s performance is in line with Grade A classification of the British Hypertension Society standard^17,53^ (Grade A if 60% of errors are ≤ 5 mmHg, Supplementary Table 7), and in line with the AAMI standard^17,53^ (Supplementary Table 8).

In software-based BP estimation methods, the inputs and the application use-case are strongly interdependent. The PPG is conveniently accessible through non-invasive means; PPG sensors have therefore been widely employed in wearables and smartwatches to estimate the heart rate and PPG. Therefore, the majority of the software-based methods estimating the BP have used the PPG signals alone^19,25,36^. While PPG/SpO_2_ is associated with BP, the addition of ECG provides important information for improved BP estimation, as exhibited by how our method compares to earlier methods^19,25,36^. This fact is also reinforced with our analysis on optimizing the number of ECG leads for BP estimation, where BP estimation performance is found to improve with increasing the number of ECG leads.

With regards to technology implementation, we found that our BP estimation method takes less than 20 milliseconds for end-to-end processing (i.e., to estimate the systolic/diastolic BP from raw physiological signals) on a PC (CPU Intel(R) Core(TM) i9-7960X CPU @ 2.80 GHz, 125 G RAM, and additional 512GB-1TB swap memory); therefore, implementing this technology in real-time on a beat-to-beat basis is quite feasible. Also, in ambulatory BP monitors, a significant proportion of the battery’s electrical energy expenditure is in inflating the cuff, maintaining its pressure, and then deflating the cuff. Software-based methods, such as the one presented here, have the additional advantage of significantly lower electrical energy usage, so they could be used for greater number of readings and/or a longer period of time.

In this study, key challenges associated with cuffless BP monitoring are also addressed (Supplementary Table 9). Unlike traditional cuff-calibrated devices, the proposed approach (which does not employ gender or race) reports actual BP estimations of intra-arterial systolic and diastolic BP. Additionally, a feature importance and accuracy analysis with the inclusion/exclusion of demographic features is employed to distinguish the influence of hemodynamic from demographic factors (Supplementary Table 10). A significant challenge in cuffless BP monitoring is the limited BP range in standard validation datasets; however, our study leverages one of the largest reported datasets, with data from 282 subjects with a broad BP range, encompassing critical care patients in various conditions—both conscious and unconscious—while mitigating noise artifacts introduced by movement and other external factors. To address the problem of noisy data, our proposed approach incorporates a hybrid noise detection approach for ECG, SpO_2_, and BP signals, aiming to enhanced performance by addressing various noise sources, including powerline noise, jitters, pacemaker spikes, signal saturation, and baseline wonder. Lastly, for a comprehensive insight into the performance of the proposed BP model, we provide the MAE across all BP ranges, demographic groups, and validation folds.

An existing limitation of our method, and most other ML-based methods, is that they are subject to dataset-shift related concerns^47^, i.e., they do not perform well when the test data sequences come from a BP range that training sequences do not sufficiently cover. It is therefore essential to have a very large training repository of physiological signals and enough sequences/samples from each BP range. Additionally, the model’s ability to adapt to external factors like stress, physical activity, or other dynamic physiological changes should be evaluated with context-annotated data. Such data could further assess BP surges (rapid increases) and dips (drops during sleep or posture changes), improving its ability to reflect real-world physiological patterns. Furthermore, the implementation and acceptance by clinical staff require the model to deliver highly interpretable results^54,55^. While the proposed RF model does offer insights into feature importance, it does not inherently explain the underlying relationships between features and predictions. Lastly, we acknowledge that exploring alternative models to RF is valuable for further research and will continue to explore alternative methods.

In summary, there is growing research and development in making medical devices more accurate, efficient, portable, convenient, interoperable^56,57^ and accessible^58^, as well as of higher prognostic value. Although technologies for out-of-office periodic BP measurement have improved over the years, they are still not convenient or comfortable. As device manufacturers and clinicians embrace the transition to promising new technologies for the continuous measuring/estimating of BP, in the advent of the artificial intelligence^54,59^ and the ever increasing use of ML/DL based methods, like the one presented here, may make it possible to routinely measure ambulatory BP in broader clinical settings, and in more diverse populations.

## Methods

### Database

The study was approved by the Institutional Review Board of Massachusetts General Hospital. Since this was a retrospective study, informed consent was waived by the ethics committee. This retrospective study was performed in accordance with the Declaration of Helsinki. Adhering to the institutional review board guidelines, we obtained de-identified data from the bed-side monitors of the intensive care units (ICUs) of Massachusetts General Hospital (MGH).

The data, ~2TB telemetry waveform data, consists of 4 channels of ECG, ABP and SpO_2_ waveforms recorded using two device manufacturers: GE Healthcare and Philips Healthcare patient monitoring systems with sampling rates of 240 Hz and 250 Hz, respectively. The ABP was collected using a gold-standard intra-arterial catheter approach, providing continuous systolic and diastolic BP. Since the devices collected data at different sampling rates, data was resampled to 250 Hz. After this step, the waveforms from all devices were treated equally. In the hybrid convolutional neural network (CNN) noise detector model, data was screened for noise across devices, therefore forcing the annotator to learn and perform well across devices. We used streaming data acquired from 282 ICU patients at MGH (~30 M beats, ~400 patient days).

To address memory constraints posed by the large dataset, the data was partitioned into smaller, manageable segments for preprocessing and training. Parallel processing techniques were employed to accelerate preprocessing and feature extraction, utilizing distributed computing. The preprocessed data and extracted features were stored in MATLAB’s .mat format. Key features from each segment were then concatenated into a single array for training, ensuring the model had access to essential data without overloading system memory.

### Patient demographics

Of the 282 patients (see Table 2), 181 were male, 99 were female, and for 2, the gender information was unavailable. With respect to race, 8 were Asian, 11 were Black, 17 were Hispanic, 214 were White, and for 32 patients, the self-declared race was unavailable. The patient population had a mean age of (66.21 ± 14.39) years and a mean body mass index (BMI) value of (28.77 ± 7.22) (see Supplementary Table 11).

**Table 2:** Summary of the dataset population by gender and race, including the number of subjects and corresponding 5-beat sequences

	Number of subjects per gender
Female	99
Male	181
Unknown	2
	Number of subjects per race
Asian	8
Black	11
Hispanic	17
White	214
Unknown	32
	Number of 5-beat sequences per gender
Female	1733298
Male	2729997
Unknown	63847
	Number of 5-beat sequences per race
Asian	102964
Black	118462
Hispanic	627470
White	3260251
Unknown	417995

### Hybrid CNN approach for noisy signal detection

We wanted to ensure that the physiological signals were noise-free. A hybrid-CNN approach was accordingly designed and trained for the purpose of detecting noise in ECG, SpO_2_, and ABP; details of the noise-detector have been provided in the Supplement (see Supplementary Fig. 17, Supplementary Table 12). Additionally, SpO_2_ and ABP were considered noisy if their values ranged outside 0–100% and 0–250 mmHg ranges, respectively.

A time-segment of physiological signals was considered noise-free only when the corresponding SpO_2_, ABP, and all 4 ECG leads were identified as noise-free, and appropriate for training/testing of our BP estimation method. This procedure of identifying noise-free segments of physiological signals was applied to the entire repository of telemetry data, and all noise-free segments were segregated for further processing. For the hybrid-CNN noise model, 80% of the data was allocated for training and validation, while the remaining 20% was reserved for testing. During testing we found that our trained hybrid-CNN model detected noise in the ECG with a sensitivity of 94.0% and specificity of 91.9%, the hybrid-CNN BP noise classifier provided a sensitivity and specificity of 88.6% and 90.9% respectively, and the hybrid-CNN SpO_2_ noise classifier provided a sensitivity and specificity of 98.5% and 94.9%, respectively^60^.

Supplementary Fig. 17 illustrates the hybrid-CNN approach for detecting noise in the ECG, SpO_2_ and ABP. Supplementary Fig. 18 displays an example of a noisy and a non-noisy ECG signal. The method was designed to process ECG, SpO_2_ and ABP signals of length 4-sec with 0.5-sec overlap, at a sampling rate of 250 Hz.

Features were extracted from the ECG, SpO_2_ and ABP using both hand-engineered signal processing techniques and convolutional layers (as used in a convolutional neural network), and finally with a fully connected network, the method determined whether the ECG signals were noisy or clean. The part containing convolutional layers had the following architecture: the first 1D-convolutional layer had 16 filters of length 500 followed by a rectified linear unit (ReLU) and 1-D max pooling layer of size 2, afterwards we had another 1D-convolutional layer with 32 filters of length 250, followed by a rectified linear unit (ReLU), and a 1-D max pooling layer of size 2. We optimized the classifier performance using the Tensorflow Keras Tuner Bayesian Optimization using the cross-entropy loss function, by varying the number of filters, filter length and pooling operations, to determine the effect of the convolution filter length as well as the network capacity, in terms of the number of trainable parameters.

Following 3 expert clinician annotation of ~80 K s of signals from different patients, as either clean or noisy, the noisy signals were found to be primarily of four types:

(i) large artifacts caused by loose contact of leads
(ii) huge baseline-wander due to patient movement
(iii) high frequency noise/jitters
(iv) flat segments and signal saturation

We designed a method to account for the above types of noise. Specifically, as a first step, the noise detector examined the presence of a flat segment or ECG, ABP and SpO_2_ saturation; if detected, the signal was ascertained as noisy. If the signal passed this step, we extracted the following hand-engineered features:

1. *Periodicity measure*^61^ measures the variability in R-R intervals. If we let vector ${\bf{I}}$ contain the R-R intervals, and ${{\rm{\sigma }}}_{I}$ and $\bar{I}$ be the standard deviation and mean of the vector, then the periodicity measure is computed as in Eq. (1):
  1 $${PM}=100-\frac{100\times {{\rm{\sigma }}}_{I}}{\bar{I}}\,$$
2. *Correlation measure*^61^ measures the mean correlation of successive QRS complexes of all beats. Clean signals are generally expected to exhibit a high correlation measure.
3. *Maximum signal energy beyond 12* *Hz*^60^ measures the maximum energy of high frequency components in the signal. The feature is computed by taking the maximum of the spectral energy contained in frequency components above 12 Hz. A higher signal energy above 12 Hz indicates poorer signal quality.
4. *Sharpness*^60^ measures how steep does the signal change around the QRS. A well-defined QRS is expected, except for abnormal cases such as ventricular fibrillation or ventricular tachycardia. Sharpness is given by $\left(\frac{2}{\pi }\right)* {\tan }^{-1}\bar{S}$; $\bar{S}$ denotes the sharpness of each QRS mean.
5. *δP Stability*^60^ measures the consistency of waveform amplitudes by assessing the difference between maximum and minimum amplitudes. Higher Stability indicates better signal quality.
6. *Peak height stability*^60^ measures the consistency of the ECG R-peaks amplitude over time. A more stable peak height indicates better signal quality. The peak height stability is given by $1-\frac{{S}_{{\boldsymbol{\delta }}{\bf{P}}}}{\overline{\overline{{\boldsymbol{\delta }}{\bf{P}}}}}$.

### Signal pre-processing and delineation

The baseline wander of the ECG signals was removed using a median filter based method^62–64^. The median filter^62^ allowed the replacement of each data point with the median of neighboring points, effectively removing outliers and preserving edges in the signal, without significantly distorting the QRS complex of the ECG signal^65^. Its performance has been compared to other methods^66,67^, with the results showing that while wavelet-based techniques and FIR filters may offer superior performance in some metrics, the median filter remains a viable option due to its simplicity and effectiveness. Unlike other methods, like spline interpolation, it does not rely on detecting the PQ interval or the precise annotation of fiducial points such as the R-wave or the P-wave, making it less prone to errors from automatic signal processing software. The presence of pacing spikes is a common concern while identifying the R-wave peaks in ECG signals. For patients with a pacemaker, a 51-order FIR filter with a 15 Hz cut-off frequency^68^ was used on the ECG to filter out the pacing spikes before the delineation process (Supplementary Fig. 19). Then, the data was calibrated using scale factors provided by the device manufacturers to convert signals into having standard units. Then, a state-of-the-art wavelet-based method by Martinez et al., ^69,70^ was used for delineating the ECG signal.

To delineate the SpO_2_ and ABP signals, we employed a modified version of Zong’s method to identify valleys^61^. This approach builds on Zong’s original open-source algorithm but introduces a few modifications. First, the low-pass filtering step was omitted, preventing artifacts and residual effects that could distort the signal; instead, a second application of the slope sum function was used to enhance sharp slopes and improve the detection of critical features in the signal. Lastly, the adaptive thresholding mechanism in Zong’s method was replaced with Martínez’s algorithm^69^, commonly utilized for QRS detection. Once the valleys in the SpO_2_ and ABP signals were identified, the maximum and minimum amplitudes between consecutive valleys were recorded and stored for subsequent feature extraction.

### Beat sequence identification and selection

We then divided the physiological signals into window segments (number of heart beats) of fixed length, wherein the input signal (ECG, SpO_2_) characteristics were extracted and used to estimate the corresponding median BP of that segment/window. With short windows, we can get more instantaneous BP estimates, but inaccuracies due to even slight disruption of the heart rhythm may affect the estimation; on the other hand, longer window segments would capture the overall signal characteristics, but the estimates would not be instantaneous. The length of the window is therefore an important parameter to optimize.

**Fig. 5:**
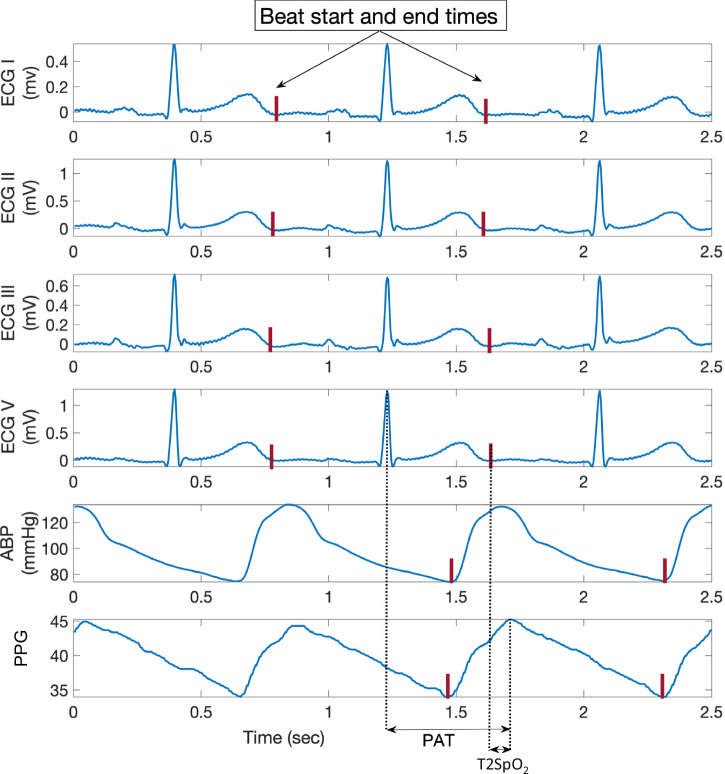
Illustration of the extent/span of a single beat in electrocardiographic, arterial blood pressure, and pulse oximetry signals. A single beat in electrocardiographic (ECG, from all 4 leads) extends from the end of T-wave of last beat to the end of T-wave of current beat. For oxygen saturation of peripheral arterial blood (SpO_2_) and arterial blood pressure (ABP), the corresponding beat extends between two consecutive valley points.

At the single beat level, the extent of a single ECG beat (in all 4 leads) was defined from the end of T-wave of prior beat to the end of T-wave of the current beat; for SpO_2_ and ABP, the corresponding beat was defined between two consecutive valley points. Figure 5 illustrates these extents.

We identified windows consisting of {1, 5, 10, 20, 30, and 50} consecutive beats from the entire noise-free data, and made sure that all sequences of beats formed contiguous segments of signals. In case the extent of a beat was not certain in all six physiological signals, the beat was dropped from the analysis, and the corresponding sequences containing such beats were also dropped. Finally, we ensured that the beat sequences used for the BP estimation were non-overlapping, i.e., no two sequences had overlapping data.

### Feature selection and estimation

All features employed in this study are presented in Supplementary Table 13; these involve time-based, spectral, statistical, and non-linear features. In this study, we build upon prior studies^71–73^ by developing and incorporating features pertinent to the BP estimation, such as the T2SpO_2,_ QT-interval duration, T-wave amplitude, Kaiser-Teager energy features, autoregressive coefficients, as well as subject-specific characteristics such as gender, body mass index and race.

Overall, for each window/sequence, we extracted 48 features from the SpO_2_ and the 4-lead ECG signals. These included the heart rate (HR), statistical measures obtained from each ECG lead (i.e., signal mobility, signal complexity, fractal dimension, entropy, and autocorrelation), duration of the corrected QT-interval and T-wave amplitude, the pulse arrival time (PAT), T2SpO_2_, entropy of the power spectral distribution of the SpO_2_, statistical measures over the Kaiser-Teager energy of the SpO_2_ signal, and coefficients of an autoregressive model fit of the SpO_2_ signal. The features were not normalized to maintain the interpretability of the physiological values.

Some features, such as the corrected QT-interval and the T-wave amplitude, were calculated on a beat-to-beat basis. For such features, if the sequence had a length greater than one beat, the median value of the feature across all beats in the sequence was used as the final value.

The minimum and maximum amplitude ABP values of each beat were identified as the gold-standard diastolic and systolic BP values of that beat. If the sequence had a length greater than one beat, the median systolic /diastolic values across all beats in the sequence were used as the gold-standard. Only those sequences that had a diastolic/systolic BP between (80–220) mmHg /(40–120) mmHg were used in our studies.

In this manner, from every sequence, one input feature vector and a corresponding gold standard estimate for systolic/diastolic BP were extracted; together, the resulting pair was termed as one data sequence from the perspective of ML.

### Features characterizing ECG morphology

We have used 7 features to characterize the ECG morphology of each of the four leads, resulting in a total of 28 features. The 7 features are explained below.

1. *Signal mobility*, measures the level of variation in the signal. If ${\boldsymbol{x}}$ denotes the ECG signal vector of length $N$, ${x}_{i}$ is the ${i}^{{th}}$ sample, and ${d}_{j}={x}_{j+1}-{x}_{j}$, is the first order variation in the signal, then ${S}_{0},$ ${S}_{1}$, and signal mobility are defined as in Eq. (2):
  2 $${S}_{0}=\sqrt{\frac{{\sum }_{i=1}^{N}{x}_{i}^{2}}{N}},{S}_{1}=\sqrt{\frac{{\sum }_{j=2}^{N}{d}_{j}^{2}}{N-1}}$$
  where, signal mobility, is defined as the ratio of ${S}_{1}$ to ${S}_{0}$.
2. *Signal complexity*, measures the second order variation in the signal (i.e. first order differences of ${\boldsymbol{x}}$, ${\boldsymbol{d}}$,). Following above notation, signal complexity is denoted as ${S}_{2}$, and is defined as in Eq. (3):
  3 $${S}_{2}=\sqrt{\frac{{\sum }_{j=2}^{N-1}{({d}_{j+1}\,-\,{d}_{j})}^{2}}{N-2}}$$
3. *Fractal dimension*, together with multi-scale entropy, measures the self-similarity of the signal. It identifies the patterns hidden in the signal at different portions and different zoom levels, and then compares them^23^.
4. *Entropy*, measures the state of disorder or randomness in the ECG signal. To compute the entropy, the entire amplitude variation (scale) of the ECG signal is divided into $M$ bins, and then the number of occurrences when the ECG sample value falls in each of the bin is computed. Dividing the number of occurrences to the total number of samples gives the probability of an ECG sample value to lie in one of those bins. Once the probability ${p}_{i}$ is computed for all bins, the entropy is computed as in Eq. (4):
  4 $${\rm{E}}{\rm{n}}{\rm{t}}{\rm{r}}{\rm{o}}{\rm{p}}{\rm{y}}=\mathop{\sum }\limits_{i=1}^{M-1}{p}_{i}{\rm{l}}{\rm{o}}{\rm{g}}\left(\frac{1}{{{\rm{p}}}_{{\rm{i}}}}\right)$$
5. *Autocorrelation*, measures the similarity/correlation of the signal with a delayed copy of itself, and helps identify repeating patterns. For extracting a single feature here, we output the autocorrelation function value at the center.
6. *QT Interval*, measures the median QT interval duration across all beats in the sequence, from the corresponding ECG lead. The QT interval encompasses the duration of ejection/systole part of the cardiac cycle^71^; hence, we believe it will be useful for BP estimation.
7. *T-wave amplitude*, measures the median T-wave amplitude across all beats in the sequence, from the corresponding ECG lead.

### Features characterizing SpO_2_ morphology

We extracted 10 features from the SpO_2_ signal. Of these, four are taken from the Kaiser-Teager energy vector, one from the power spectral distribution of the SpO_2_ signal, and five from an auto-regressive model which is made to fit over the SpO_2_ signal.

1. *Kaiser-Teager energy (KTE)*, is a popular tool used in signal processing for finding the energy profiles of signals with periodic components, and for end-point detection. If ${\boldsymbol{s}}$ is the vector containing the SpO_2_ signal, ${s}_{i}$ denotes the ${i}^{{th}}$ sample of the SpO_2_ signal, and vector ${\boldsymbol{KTE}}$ is the Kaiser-Teager energy of the SpO_2_ signal, then KTE is computed as in Eq. (5):
  5 $${KT}{E}_{i}={s}_{i}^{2}-{s}_{i+1}.{s}_{i-1}$$
  The ${\boldsymbol{KTE}}$ vector has unique properties. If the SpO_2_ is a periodic signal (signal made of periodic components), the mean value of the ${\boldsymbol{KTE}}$ vector is high, and if the SpO_2_ signal is noisy, the mean value of the ${\boldsymbol{KTE}}$ vector is low. Four features are extracted from the ${\boldsymbol{KTE}}$ vector, namely the mean of the vector (${KT}{E}^{\mu })$, the variance (${KT}{E}^{\sigma })$, the inter-quartile range (${KT}{E}^{{iqr}}$), and skewness (${KT}{E}^{{skew}}$).
2. *Spectral entropy*, the power spectral distribution of SpO_2_ signal is computed using the Fast Fourier Transform. We then compute the entropy of the power spectral distribution and utilize it as one of our features; this feature is called spectral entropy. Clean signals are expected to have low spectral entropy, while noisy signals (that may include a flat or tilted power spectrum) are expected to have high spectral entropy value.
3. *AR coefficients*, the coefficients learned while training an autoregressive (AR) model over the SpO_2_ signal, can provide useful information on the propagation of the ECG signal through arteries, veins and capillaries^72^. The AR coefficients appear to model the spectral envelope of the SpO_2_ signal and also model the basic shape of the pulse. For our approach, we train a 5th-order AR model over the SpO_2_ signal, and the five coefficients learned from the signal are used as features.

### Other features

1. *Heart rate*, the median heart rate of the patient as measured in the given window, was utilized as a feature. The heart rate was measured by identifying the median R-R interval across all 4-leads of the ECG and across all beats. For windows of length one beat, the instantaneous heart rate, as measured by the R-R interval between R-wave peak of current beat to that of the last beat, was used^73^.
2. *Pulse Arrival Time (PAT)*, is expected to give an indication of the pulse propagation velocity within arteries. It involves the detection of blood pulse at different arterial sites, and then measures the respective time delays. There are numerous studies^24,74^ which have indicated a strong relationship between PAT and BP. Here, we measure PAT as the mean time delay between the ECG signal’s R-wave peak (onset of pulse) to the corresponding peak observed in the SpO_2_ signal (which is measured at fingers, ears etc). PAT has been depicted in Fig. 5.
3. *T2SpO*_*2*_ is measured by taking the duration between the peak of SpO_2_ and the end of T-wave of the corresponding beat. T2SpO_2_ is depicted in Fig. 5.

### Patient characteristics

We also included patient specific details as features/inputs for BP estimation; these details were retrieved from their medical records. The features are:

1. *Age* of the patient^75^.
2. Body mass index (BMI) of the patient^76^.
3. Gender of the patient. This variable was treated as a categorical variable. Value of “1” was used to denote “female”, “2” was used to denote “male”, and “NaN” was used to denote the case where either the gender of the patient was unavailable or when it was different from male and female^75^.
4. Race of the patient. The self-declared race was also treated as a categorical variable. The races considered were: Asian, Black, Hispanic and White. To input this information to ML models, race was encoded as a feature vector of size four, in a way similar to one-hot vector encoding. If the race was “White”, the four sized feature vector would be [1, 0, 0, 0]; if race was “Hispanic” the vector would be [0, 1, 0, 0]; if race was “Asian” the vector would be [0, 0, 1, 0], and if race was “Black” the vector would be [0, 0, 0, 1].

### Random forest (RF) based regression model and performance assessment

Once the input features and gold-standard output were extracted, we proceeded towards fitting an RF based regression model. For this study, we used the Treebagger function from MATLAB 2018a, which is MATLAB’s implementation of the RF algorithm^77^. For a small and simple search space, such as optimizing a single feature with discrete values, a grid search was preferred due to its simplicity and interpretability. The computational cost of the grid search in this context was manageable, and it allowed for a comprehensive understanding of how the number of trees—and thus model complexity—impacted performance.

We built separate RF regression models for estimating the systolic and the diastolic BP. Feature selection was performed using Random Forest’s Out-Of-Bag (OOB) samples to measure each feature’s importance based on how much the prediction error increased when a feature’s values were randomly permuted, while using curvature tests to select features for splitting nodes in the decision trees. Information on the RF structure and hyperparameters can be found in Supplementary Table 14.

To assess the BP estimation performance, we trained/tested the ML method using five-fold cross-validation. Since all sequences were non-overlapping, this ensured that no overlap existed in the train and test data of the five folds. A five-fold cross-validation approach was utilized to determine the random forest model parameters, including the optimal number of trees, window length (number of beats as input), feature selection, and the number of leads, as well the final evaluations for all models, including the patient-specific model, where for each test-set fold, a model is trained with the patient data also divided in five-folds. This method provided a balance between computational efficiency and robust evaluation by testing across multiple test sets, reducing the risk of overfitting to a single data partition and ensuring reliable performance estimation.

### Statistical methods

Continuous data are presented as mean and standard deviation, median and inter quartile ranges are used in the box plots. We used the linear mixed model (LMM) to assess the significance of observed differences between groups, using a significance level of 0.01. To meet the normality assumption of an LMM, a log transformation was used to approximate the residuals to a normal distribution, and p-values were calculated to assess statistical significance.

We conducted all statistical analyses using MATLAB (Version: R2018a) and Python (Package: StatsModels; Version: 0.14.4).

## Supplementary information

OnlineSupplement_unmarked

## Data Availability

The data will be available to any investigator upon request.

## Code availability

The code will be available to any investigator upon request.
