# Supplementary material for: Machine learning based, subject-specific, gender and race independent, non-invasive estimation of the arterial blood pressure
Source: NPJ Cardiovasc Health. 2025 Aug 1;2:41. doi: 10.1038/s44325-025-00075-5 (PMC12316593; doi:10.1038/s44325-025-00075-5)

Machine Learning Based, Patient Specific, Gender and Race Independent, Non-invasive Estimation of the Arterial Blood Pressure

Rahul Kumar Sevakula, Patrícia J. Bota, Mohamad B Kassab, Sandeep Chandra Bollepalli, Geerthy Thambiraj, Richard Boyer, Eric M. Isselbacher, Antonis A. Armoundas

Identification of the Optimal Number of Trees in Random Forest (RF) Regression Model

We measured the 5-beat sequence Blood Pressure (BP) estimation performance using 200, 400, 600, 1000 trees respectively, and the results are presented in Supplementary Table 2.

We understand that it is necessary for the regression methods to be robust and computationally efficient. By increasing in the number of trees, the RF models are theoretically becoming more robust, however their computational requirements also increase. In Supplementary Table 2, we observe that the 600 tree RF model is computationally demanding but provides very little benefit in performance, compared to the 400 one; the 200 tree RF model provides similar performance with the 400 tree RF model, but considering that the RF model is being trained over ~3.6 M samples in each of the five-folds, 200 trees may not be enough to take the full-benefit of bagging variations[1]. Therefore, we choose the 400 trees model as the one providing sufficient balance of being robust and computationally efficient.

Identification of Feature Importance in Random Forest Regression Model

RF regression models are designed with the ability to judge the importance of individual features during regression. In the Treebagger implementation of RF, feature importance scores are computed by summing the changes in the nodes' risk due to splits on the individual feature, and then dividing the sum by the number of branch nodes. Here, risk is defined as the product of the node's probability to the mean squared error at the node, and change in node risk, is defined as the difference between the parent node's risk to the sum-total risk of children nodes. A higher feature importance score indicates a better feature. For BP estimation, we derived the mean importance of each feature with the RF models trained in each of the five folds. For purposes of this ranking, the features were sorted in descending order of their mean importance. Since different RF models were independently trained for estimating the systolic and diastolic BP, the corresponding ranking of features remained different, as well.

Supplementary Table 3 presents the feature ranking obtained by RF models trained over 5-beat non-overlapping sequences. Supplementary Figures 1 and 2 presents box plots of feature importance scores across five-fold, during systolic BP estimation and diastolic BP estimation, respectively. It appears that features pertaining to gender, race, fractal dimension from ECG, and coefficients of the autoregressive model fitted over SpO_2, are of significantly less importance; hence, they were eliminated in the final BP estimation model, and the final model was trained using only 34 selected features.

Impact of the Demographic Features on Model Performance

In Supplementary Table 10 of the Online Supplement, we examined the impact of including and excluding key demographic features (HR, BMI, and age), identified as top contributors in the feature selection process, on the model's performance. The experimental results showed that for all three models (primary, subject-specific, augmented subject-specific) using only demographic features (age, BMI, and HR) results in lower performance compared to the case that all selected features were employed, indicated by an increase in MAE, for both systolic and diastolic BP estimations (p-value < 0.01, LMM). A similar trend was observed when we excluded age and HR features only (p-value < 0.01, LMM). In contrast, omitting BMI, does not

affect the model' performance for the subject-specific model, whereas the primary and augmented models slightly improve their accuracy (p-value < 0.01, LMM).

These results confirm that the model performance primarily depends on hemodynamic characteristics rather than individual demographic features.

Performance evaluation with different number of leads and lead combinations

As explained in the main manuscript, we assessed the BP estimation performance using different number/combinations of ECG leads together with the SpO_2 signal. From the four ECG leads, lead, 1, 2 and 3 were limb leads while lead 4 was a precordial lead. Supplementary Table 4, below, presents the MAE during systolic/diastolic BP estimation employing different lead combinations. BP in all experiments, was estimated with the 34 selected features.

Identification of the Optimal Number of Trees for Patient-Specific RF Regression Models

To determine the number of trees for patient-specific RF models, we measured the BP estimation performance with the primary RF model, the patient-specific model, and the augmented patient-specific model, while changing the number of trees in patient-specific RF models across {50, 100, 200, 300, 500}, and the results are presented in Supplementary Table 5.

There are a few important observations regarding the number of trees in patient-specific RF model, as follows: (i) since the number of data samples available for training would be relatively few in number, fewer number of trees would suffice, (ii) the number of trees in the patient-specific RF model determines whether the estimates of the augmented patient-specific model would lean towards the primary RF model or towards the patient-specific model estimates. In Supplementary Table 5, one observes that there is no performance improvement in the patient-specific models after 100 trees; furthermore, with 100 trees, the augmented patient-specific RF model shall leans more towards the primary RF model, which is satisfactory from the point of view of being immune to dataset-shift related issues pertaining to the patient-specific RF model. Therefore, we choose 100 trees to be an appropriate number for the patient-specific RF models.

Principles of Blood Pressure Measurement and Estimation

Two primary approaches have been identified for measuring the blood pressure (BP): cuff-based and cuffless devices[2]. The traditional cuff-based method involves inflating a cuff (manual or automated) around the upper arm or wrist, to temporarily occlude blood flow and gradually deflating it while measuring the pressure exerted by the blood against the arterial walls. The cuff-based BP measurement is today's main practice in office readings. However, this one-shot measure has several limitations, failing to capture dynamic variations influenced by daily activities and making it impossible to reproduce BP readings[3].

Office readings shortcomings include the reliance on a limited number of data points (measurements taken every few minutes)[3]; discomfort associated with cuff inflation leading to infrequent use[2]; brachial cuff BP measures underestimate intra-arterial systolic BP and overestimate intra-arterial diastolic BP by 5 to 6 mmHg[3,4]; and susceptibility to variability due to factors such as the white-coat effect (15-25% elevated office BP levels but normal out of the office BP levels[3,5,6]), masked hypertension (normal BP value in the office but elevated outside the office in 10 to 20% individuals[3,6]), and setting (being alone or in office setting) and procedural influences (resting, talking, arm lower than heart level, unsupported arm, legs crossed, how many measures are considered)[3].

Given these challenges, out-of-office monitoring, such as 24-hour ambulatory BP measurement, is recommended. However, these methods also exhibit high variability and are underutilized due to the discomfort associated with the use of the cuff .

Cuffless BP devices have emerged as a promising alternative, allowing for multiple and continuous measurements during routine activities without the discomfort of cuff-induced limb compression[3]. These devices leverage various technologies, including pulse transit time (PTT), pulse wave analysis (PWA), and facial video processing, to estimate BP without requiring traditional cuffs[2]. The diversity in methods often results in different brands providing diverse BP readings[3,7], making it difficult to evaluate the device's accuracy with universal evaluation standards.

Cuffless devices can be divided in: (1) Cuff-Calibrated Devices: these devices require periodic calibration using an oscillometric upper-arm cuff device or the subject's demographic information[2,3]; (2) Calibration-Free Devices: these devices do not require periodic recalibration and rely on advanced signal processing techniques.

Despite their potential benefits, cuffless BP measurement devices face several challenges (see Supplementary Table 9). One major challenge involves existing validation standards, which have been developed for cuff-based devices and are not adequately accounting for the unique characteristics of cuffless technologies[2,7]. Cuffless calibrated devices track BP changes relative to the initial calibration measure, with their accuracy being dependent on periodic recalibration. Thus, they are difficult to maintain and assert their accuracy over time[7]. Second, the devices 'performance is often aggregated at a population level, which can obscure individual variability and lead to an overestimation of a device's true accuracy for a specific user. Therefore, a comprehensive validation requires the analysis over a diverse population with both inter and intra-individual BP changes induced by a complete set of BP-related physiological mechanisms[7]. Thirdly, cuffless devices often incorporate demographics information, which the literature has shown to correlate with BP[7,8], and display their results without clearly showing if the good correlations result from the demographic or the hemodynamic characteristics[7].

Lastly, calibration-free devices often rely on physiological signals, which are inherently noisy due to motion, sensor displacement, environmental noise, among others.

To address these challenges, subject-specific performance metrics such as mean absolute error (MAE) and standard deviation of errors should be utilized to evaluate performance across diverse users[7]. Furthermore, the inclusion of demographic and biometric data has shown potential to improve the accuracy of BP estimations[3,7].

In summary, traditional clinical approaches, which often rely on subjective evaluations and a limited number of data points, are increasingly being surpassed by AI-driven methods[9]. Cuffless BP monitoring presents a promising solution for large-scale clinical measurements, enabling early diagnosis and intervention of asymptomatic conditions. Unlike traditional methods, cuffless devices provide continuous and long-term monitoring, offering a more reliable

estimate of BP fluctuations over time compared to in-office measurements[3]. Moreover, they have the potential to increase patient compliance by offering daily feedback on BP trends and health evolution.

Online Supplement Tables

Supplementary Table 1 Features and performance of other models used towards blood pressure estimation. PPG: Photoplethysmography; SVM: Support Vector Machine. PIR: Photoplethysmography intensity ratio; PTT: Pulse Transit Time; DNN: Deep Neural Network; Sys: Systolic, Dias: Diastolic, MAE: Mean Absolute Error; RMSE: Root Mean Squared Error; RF: Random Forest. Best performing method and performance is shown.

Study	Data	Signals	Model	Features	Performance (mmHg)
Zhang et al.[10]	7,000 beats (from the University of Queensland Vital Signs Dataset) Non-invasive ABP	PPG	SVM	Systolic upstroke Time, Diastolic Time, Diastolic width at 25%, 50%, 75%, Systolic width + Diastolic width at 25%, 50%, 75%, from PPG signal	Sys MAE: 11.64 ± 8.20<br>Dias MAE: 7.62 ± 6.78
Radha et al.[11]	17 subjects, 1,670 beats. Non-invasive BP	ECG and PPG	RF	Pulse wave velocity calculated with pulse arrival time found from ECG and PPG signals	Sys RMSE: 7.86 ± 1.57<br>Dias RMSE: 6.75 ± 1.41
Ding et al.[12]	33 subjects, 5,024 beats. Non-invasive BP	PPG	PTT-PIR Equation	Pulse transit time (PTT) and PPG intensity ratio (PIR), from PPG signal	Sys MAE 1.17 ± 5.72<br>Dias MAE: 0.46 ± 5.49
Slapnicar et al.[13]	510 subjects, ~29 patient days (from MIMIC. Invasive ABP)	PPG	ResNet	Cycle duration time, time from cycle start to systolic peak, time from systolic peak to cycle end, time from cycle start to first peak in PPG' (steepest point), time from cycle start to second peak in PPG' (dicrotic notch), time from systolic peak to dicrotic notch, time from dicrotic notch to cycle end, ratio between systolic and diastolic amplitude frequency, three most dominant frequencies and their corresponding power spectral density amplitude. Features generated with	Sys MAE 9.43<br>Dias MAE: 6.88

				deep learning architectures were also used.	
Simjanoska et al.[14]	51 subjects, 3,129 segments of 30 sec length (Invasive and non-invasive ABP)	ECG	Stacked-based calibrated (KNN, J48, Naïve Bayes, SVM, RF, Bagging, Boosting)	Signal mobility, signal complexity, fractal dimension, entropy, autocorrelation of ECG signal, and age	Sys MAE: 7.72<br>Dias MAE: 9.45
Kachuee et al.[15]	942 subjects, 3,663 records (length not provided) from MIMIC II. Invasive radial ABP	ECG and PPG	Calibrated AdaBoost	PAT, HR, augmentation, large artery stiffness, inflection point area ratio, whole based features	Sys MAE: 8.21<br>Dias MAE: 4.31
Wang et al.[16]	90 subjects, ~200 patient days (MIMIC). Invasive ABP	PPG	DNN	Systolic upstroke time, diastolic time, spectral features using multitaper method	Sys MAE: 4.02 ± 2.79<br>Dias MAE: 2.27 ± 1.82
**This study**	**282 subjects, ~400 patient days**	**ECG and SpO_2**	RF	Pulse arrival time, heart rate, statistical parameters of Teager Kaiser energy profile of SpO_2 signal, signal mobility, signal complexity, fractal dimension, entropy, autocorrelation, QT intervals, and T-amplitudes of ECG signal, age and BMI	Sys MAE: 4.29 ± 5.00<br>Sys RMSE: 6.60 ± 0.01<br>Dias MAE: 2.38 ± 3.25<br>Dias RMSE: 4.04 ± 0.02

Supplementary Table 2 BP estimation performance with 5-beat sequences, before feature selection.

# Trees in RF	MAE in estimating systolic BP (mmHg)	MAE in estimating diastolic BP (mmHg)
200	4.55 ± 5.26	2.71 ± 3.62
400	4.55 ± 5.26	2.70 ± 3.62
600	4.54 ± 5.26	2.70 ± 3.63
1000	4.54 ± 5.26	2.70 ± 3.63

Supplementary Table 3 Feature ranking in blood pressure estimation. The ranking was obtained by taking the mean relative ranking of each feature, as ascertained by the five RF models trained over 5-beat non-overlapping windows.

Feature Rank	Systolic BP Estimation	Diastolic BP Estimation
1	T-amplitude, lead 4	age
2	signal mobility, lead 4	bmi
3	age	signal complexity, lead 4
4	T-amplitude, lead 2	autocorrelation, lead 1
5	heart rate	signal mobility, lead 4
6	signal mobility, lead 2	T-amplitude, lead 4'
7	QT interval, lead 2	autocorrelation, lead 2
8	signal mobility, lead 3	signal complexity, lead 1
9	QT interval, lead 1	autocorrelation, lead 3
10	bmi	autocorrelation, lead 4
11	entropy, lead 3	QT interval, lead 3
12	signal complexity, lead 1	PAT
13	autocorrelation, lead 1	T-amplitude, lead 2'
14	autoregressive model coefficient 3	QT interval, lead 1
15	signal complexity, lead 3	T-amplitude, lead 3
16	QT interval, lead 4	QT interval, lead 4
17	autoregressive model coefficient 2	signal mobility, lead 1
18	autocorrelation, lead 3	signal complexity, lead 3
19	PAT	signal mobility, lead 2
20	T-amplitude, lead 3	QT interval, lead 2
21	T-amplitude, lead 1	$T2SpO_2$
22	autocorrelation, lead 4	entropy, lead 4
23	entropy, lead 1	signal mobility, lead 3
24	$T2SpO_2$	autoregressive model coefficient 3
25	signal mobility, lead 1	signal complexity, lead 2
26	QT interval, lead 3	heart rate
27	KTE interquartile range	T-amplitude, lead 1
28	signal complexity, lead 2	entropy, lead 1
29	autocorrelation, lead 2	entropy, lead 3
30	KTE variance	KTE variance
31	signal complexity, lead 4	entropy, lead 2
32	fractal dimension, lead 3	KTE interquartile range

33	fractal dimension, lead 4	autoregressive model coefficient 2
34	entropy, lead 2	KTE mean
35	KTE mean	KTE skewness
36	fractal dimension, lead 1	race, hispanic
37	race, african american	spectral entropy
38	race, hispanic	fractal dimension, lead 4
39	autoregressive model coefficient 5	fractal dimension, lead 3
40	autoregressive model coefficient 4	autoregressive model coefficient 1
41	spectral entropy	fractal dimension, lead 1
42	autoregressive model coefficient 1	race, african american
43	fractal dimension, lead 2	gender
44	entropy, lead 4	fractal dimension, lead 2
45	gender	autoregressive model coefficient 5
46	race, white	race, asian
47	KTE skewness	race, white
48	race, asian	autoregressive model coefficient 4

Supplementary Table 4 Blood pressure estimation performance using SpO_2 and ECG from different lead combinations. Here, leads {'1', '2', '3'} refer to the limb leads and lead '4' refers to a precordial lead. In all cases, BP has been estimated with the 34 selected features.

ECG Lead Combinations (SPO_2 is always used)	MAE of Systolic BP estimation (mmHg)	MAE of Diastolic BP estimation (mmHg)
1,2,3,4	4.29 ± 5.00	2.38 ± 3.25
1,2,4	4.36 ± 5.09	2.43 ± 3.29
1,2,3	4.59 ± 5.29	2.51 ± 3.34
1,3,4	4.37 ± 5.07	2.42 ± 3.29
2,3,4	4.42 ± 5.16	2.44 ± 3.30
1,2	4.74 ± 5.45	2.59 ± 3.41
1,3	4.79 ± 5.49	2.59 ± 3.42
1,4	4.52 ± 5.25	2.49 ± 3.34
2,3	4.85 ± 5.54	2.62 ± 3.44
2,4	4.59 ± 5.36	2.53 ± 3.38
3,4	4.56 ± 5.28	2.51 ± 3.38
1	5.21 ± 5.93	2.78 ± 3.59
2	5.32 ± 6.04	2.85 ± 3.64
3	5.33 ± 6.02	2.83 ± 3.64
4	4.96 ± 5.73	2.70 ± 3.55

Supplementary Table 5 Performance evaluation of BP estimation of the primary model, the patient-specific model, and the augmented subject-specific model, across varying number of trees.

Patient-Specific RF model # Trees	BP type	Primary Model MAE (mmHg)	Subject-Specific Model MAE (mmHg)	Augmented Subject-Specific Model MAE (mmHg)
50	Systolic BP	4.29 ± 5.00	3.56 ± 4.26	4.18 ± 4.84
	Diastolic BP	2.38 ± 3.25	1.90 ± 2.61	2.27 ± 3.03
100	Systolic BP	4.29 ± 5.00	3.51 ± 4.24	4.10 ± 4.72
	Diastolic BP	2.38 ± 3.25	1.85 ± 2.60	2.27 ± 3.00
200	Systolic BP	4.29 ± 5.00	3.52 ± 4.22	3.98 ± 4.56
	Diastolic BP	2.38 ± 3.25	1.88 ± 2.60	2.19 ± 2.87
300	Systolic BP	4.29 ± 5.00	3.52 ± 4.22	3.90 ± 4.46
	Diastolic BP	2.38 ± 3.25	1.88 ± 2.59	2.13 ± 2.79
500	Systolic BP	4.29 ± 5.00	3.51 ± 4.22	3.79 ± 4.36
	Diastolic BP	2.38 ± 3.25	1.88 ± 2.60	2.06 ± 2.70

Supplementary Table 6 Five-fold cross validation results for the primary, subject-specific and augmented subject-specific model. The table presents the mean absolute error (MAE), mean error (ME), and root mean squared error (RMSE); mean ± standard deviation.

Model	BP type	MAE (mmHg)	ME (mmHg)	RMSE (mmHg)
Primary	Systolic	4.29 ± 5.00	0.03 ± 6.60	6.60 ± 0.01
	Diastolic	2.38 ± 3.25	-0.04 ± 4.04	4.04 ± 0.02
Subject-Specific	Systolic	3.51 ± 4.24	-0.12 ± 5.52	5.52 ± 0.0
	Diastolic	1.85 ± 2.60	-0.04 ± 3.22	3.22 ± 0.0
Augmented Subject-Specific	Systolic	4.10 ± 4.72	0.00 ± 6.25	6.25 ± 0.01
	Diastolic	2.27 ± 3.00	-0.04 ± 3.77	3.77 ± 0.01

Supplementary Table 7 Models' performance in arterial blood pressure estimation compared to the British Hypertension Society standard. For each model, the percent of absolute errors, for systolic and diastolic BP that is below each of the three thresholds (<5 mmHg, <10 mmHg, and <15 mmHg), is reported. The British Hypertension Society (BHS) standard[15,17] is met when the percentage of absolute errors for the BHS thresholds (<5 mmHg, <10 mmHg, and <15 mmHg), exceeds a predefined percentage thresholds for each Grade Grade A: 60%, 85%, 95%;, Grade B: 50%, 75%, 90%; Grade C: 40%, 65%, 85%, respectively]. Results above the BHS Grade A standard are highlighted in bold. The results showed that all of our proposed models attain a Grade. A classification due to exceeding the required 60% cumulative error within ± 5 mmHg, along with the thresholds for ± 10 mmHg and ± 15 mmHg.

**Model Comparison Against the BHS Standard**				
		**Percentage of Absolute Error**		
		**< 5 (mmHg)**	**< 10 (mmHg)**	**< 15 (mmHg)**
**Primary**	Systolic	**71.86**	**90.36**	**95.86**
	Diastolic	**88. 23**	**96.96**	**98.92**
**Subject-Specific**	Systolic	**77.65**	**93.31**	**97.58**
	Diastolic	**92.43**	**98.50**	**99.48**
**Augmented**	Systolic	**73.00**	**91.00**	**96.38**
	Diastolic	**89.10**	**97.58**	**99.18**
**BHS Standard Grade A**		60	85	95
**BHS Standard Grade B**		50	75	90
**BHS Standard Grade C**		40	65	85

Supplementary Table 8 The models' performance in estimating the blood pressure is compared against the Advancement of Medical Instrumentation (AAMI) standard. The mean error (ME) and standard deviation (STD) of systolic and diastolic BP estimation are reported. According to the AAMI standard[15,17], the model meets the criteria if ME is below 5 mmHg and STD is below 8 mmHg. Results above the AAMI standard are highlighted in bold.

**MODEL COMPARISON AGAINST THE AAMI STANDARD**				
		**ME < 5 mmHg**	**STD < 8 mmHg**	**Subjects**
**Primary**	Systolic	**0.03**	**6.60**	**282**
	Diastolic	**-0.04**	**4.04**	
**Subject-Specific**	Systolic	**-0.12**	**5.52**	
	Diastolic	**-0.04**	**3.22**	
**Augmented**	Systolic	**0.00**	**6.25**	
	Diastolic	**-0.04**	**3.77**	

Supplementary Table 9 Summary of cuffless BP measurement Best Practices, Gaps and and Challenges, as well as Recommendations.

**Best Practices**	**Description**
1. **Standardized Measurement Protocols**	Ensures consistent readings across clinical and home settings
2. **Continuous Measurement**	Performance of 24-hour ambulatory blood pressure (BP) monitoring allows to capture BP variations beyond office measurements
3. **Home Monitoring**	Reduces white-coat effect while providing longitudinal BP data for better management
4. **Automated devices versus Manual Analysis**	Automated readings allow to reduce human error and improve accuracy
5. **EHR Information**	The use of demographic and biometric data from electronic health records (EHR) to improve BP estimation accuracy
6. **Use of AI Personalized Models**	Analysis of large amounts of data allows to identify hidden patterns and provide personalized models
**Gaps & Challenges**	**Background**
1. **Cuff Inflation**	Highly uncomfortable resulting in infrequent use, limiting the availability of real-time BP data
2. **Snapshot Nature of Office Measurements**	Fails to capture dynamic variations caused by stress, meals, posture, physical activity, and other physiological and behavior factors
3. **White-coat Effect**	White-coat effect increases BP levels
4. **Masked Hypertension**	Normal BP value in the office but elevated outside the office
5. **Diverse BP Estimation Methods & Readings**	Diverse sensor methods from applanation tonometry, oscillometry and ultrasonography result in highly heterogeneous, non-standardized measures
6. **Accuracy**	Existing BP validation standards have been designed for cuff-based devices and are not fully applicable to cuffless technologies, hindering clinical adoption
7. **Calibration**	Cuff-calibrated devices depend on periodic recalibration, making it difficult to maintain consistent accuracy over time
8. **BP Variability**	Individual variability in BP, affected by physiological factors (meal ingestion, alcohol or caffeine intake, bladder extension, cold exposure), postural (standing or lying), setting (being alone or in office setting) and procedural factors(resting, talking, arm lower than heart level, unsupported arm, legs crossed, how many measures are considered)

9. **Pressure Amplification**	Aortic systolic BP levels are lower than brachial BP levels. The aorta is more elastic and absorbs some of the force of the pulse wave, while reflection and arterial stiffness in the peripheral arteries result in BP amplification
10. **Cuff-based Measurements vs Intra-arterial**	Estimation value only of arterial site, quantification of mechanical stress that BP exerts in segments of the arterial tree can only be obtained with insertion of intra-arterial catheter
11. **Noisy Signals**	Adapt or remove noisy signals due to motion, environmental noise, poor sensor placement, among others
12. **Demographic vs Hemodynamic Features**	Cuff-less AI methods often include demographic (known to correlate with BP) without clearly displaying the added value of hemodynamic features
**13. Fiducial point's identification**	Incorrect valley and peak detection can lead to measurement errors
**14. Vasomotor Changes**	SpO_2 signals are sensitive to pressure variations between the skin and sensor. Vascular tone fluctuations, influenced by medications such as vasopressors, can alter SpO_2 readings, while beta-blockers may affect ECG signal interpretation
**15. Dampening of Arterial BP**	A damping effect is observed in prolonged BP measurement from an arterial catheter. Factors such as clot formation, arterial vasospasm, air entrainment, or loose connections can reduce the oscillatory amplitude of the arterial BP waveform.
**RECOMMENDATIONS**	
1. **Device Form Factor**	Develop more comfortable, user-friendly cuff designs to reduce discomfort and encourage more frequent monitoring
2. **Long-term Information**	Implement automated, remote BP monitoring solutions to provide better long-term data for clinicians
3. **Validation Standards**	Subject-specific performance metrics such as mean absolute error (MAE) and standard deviation of errors should be provided to enhance device evaluation across diverse users
4. **Demographic Information**	Inclusion of demographic and lifestyle data has been shown to increase the performance of BP estimation algorithms
5. **Big Data**	The use of a large, diverse population datasets has been shown to improve the accuracy and generalizability of cuffless devices
6. **Diverse Data Sources**	Enhance interoperability standards to streamline data integration from diverse data sources
7. **Inter and Intra-Individual BP Estimation**	Subject-specific performance metrics such as the MAE and standard deviation of errors, should be utilized to evaluate performance across diverse users

8. **Results Visualization**	Box-plots with performance across BP ranges, folds, demographic information, correlation plot and bland-Altman plot can provide comprehensive insight into the model performance

Supplementary Table 10 Impact of demographic features on model performance (mean absolute error (MAE), mmHg and p values). The effect of excluding/including Age, BMI, and HR on systolic and diastolic ABP estimation is displayed for all models. The p-values (shown between parenthesis), derived from linear mixed models statistical analysis, are presented after applying a Box-Cox transformation to ensure a normal distribution of the data. A linear mixed model was employed to account for multiple samples from the same subject.

Impact of Demographic Features on Model Performance (MAE, mmHg)						
		**Without Age**	**Without BMI**	**Without HR**	**Only Age, BMI and HR**	**All Selected Features**
**Primary**	Systolic	4.35 ± 5.01 (<0.01)	4.14 ± 4.86 (<0.01)	4.33 ± 5.03 (<0.01)	10.55 ± 9.06 (<0.01)	4.29 ± 5.00
	Diastolic	2.47 ± 3.44 (<0.01)	2.13 ± 2.78 (<0.01)	2.40 ± 3.25 (<0.01)	5.07 ± 4.83 (<0.01)	2.38 ± 3.25
**Subject-Specific**	Systolic	3.54 ± 4.24 (<0.01)	3.54 ± 4.24 (0.007)	3.59 ± 4.27 (<0.01)	10.96 ± 9.29 (<0.01)	3.51 ± 4.24
	Diastolic	1.88 ± 2.59 (0.013)	1.88 ± 2.59 (0.015)	1.91 ± 2.61 (<0.01)	5.13 ± 4.79 (<0.01)	1.85 ± 2.60
**Augmented Subject-Specific**	Systolic	4.15 ± 4.72 (<0.01)	3.99 ± 4.63 (<0.01)	4.15 ± 4.75 (<0.01)	10.56 ± 9.03 (<0.01)	4.10 ± 4.72
	Diastolic	2.10 ± 2.74 (<0.01)	1.97 ± 2.63 (<0.01)	2.09 ± 2.71 (<0.01)	5.00 ± 4.69 (<0.01)	2.27 ± 3.00

Supplementary Table 11 Summary with dataset distribution information. Data are presented as mean ± standard deviation.

**Age (years old)**	66.21 ± 14.39
**BMI**	28.77 ± 7.22
**Diastolic BP (mmHg)**	56.42 ± 9.80
**Systolic BP (mmHg)**	116.65 ± 19.60

Supplementary Table 12 Features employed in the noise detector, their brief description, and their associated signal source. Features are extracted for all ECG leads.

Index	Name	Description	Signal Source
1	Periodicity	Variability in R-R intervals	ECG, SpO_2, ABP
2	Correlation	Mean Correlation of successive QRS complexes	ECG, SpO_2, ABP
3	Max Signal Energy beyond 12 Hz	Maximum energy in the frequency components above 12 Hz.	ECG
4	Sharpness	Signal steepness around QRS complex	ECG
5	Peak height stability	Consistency of the R-peak amplitude over time	ECG
6	δP Stability	Consistency between the maximum and minimum signal amplitude difference	SpO_2, ABP

Supplementary Table 13 Features employed in the blood pressure estimation model, a brief description, and their signal associated source.

Index	Name	Description	Signal Source
1	ECG_SpO_2_PAT_Median	Median of Pulse Arrival Time, indicating time between heart activity and peripheral pulse	ECG, SpO_2
2	ECG_SpO_2_T2SPO_2_median	Median of Pulse Transit Time, measuring arterial pulse wave travel time	ECG, SpO_2
3	ECG median HR	Median heart rate, representing average cardiac activity	ECG
4	SpO_2_KTE_mean	Mean of Kaiser-Teager of the signal	SpO_2
5	SpO_2_KTE_var	Variance of Kaiser-Teager, indicating signal fluctuation	SpO_2
6	SpO_2_KTE_inter-quartile	Interquartile range of Kaiser-Teager, showing signal spread	SpO_2
7	SpO_2_KTE_skew	Skewness of Kaiser-Teager distribution, indicating asymmetry	SpO_2
8	SpO_2_Spectral_Entropy	Spectral entropy, measuring signal complexity in the frequency domain	SpO_2
9	SpO_2_AR_Feature_1	First auto-regressive (AR) model coefficient, capturing signal dynamics	SpO_2
10	SpO_2_AR_Feature_2	Second auto-regressive (AR) model coefficient	SpO_2
11	SpO_2_AR_Feature_3	Third auto-regressive (AR) model coefficient	SpO_2
12	SpO_2_AR_Feature_4	Fourth auto-regressive (AR) model coefficient	SpO_2
13	SpO_2_AR_Feature_5	Fifth auto-regressive (AR) model coefficient	SpO_2
14	ECG_{LEAD}_Signal_Mobility	Signal mobility for ECG{LEAD}, indicating signal variation	ECG
15	ECG_{LEAD}_S2	A specific statistical feature (S2) from ECG{LEAD}	ECG
16	ECG_{LEAD}_Fractal_Dimension	Fractal dimension of ECG{LEAD}, quantifying signal complexity	ECG
17	ECG_{LEAD}_Entropy	Entropy of ECG{LEAD} signal, measuring randomness	ECG

18	ECG_{LEAD}_Auto-Correlation	Autocorrelation of ECG{LEAD} signal, indicating periodicity	ECG
19	ECG_{LEAD} median_QT_interval	Median QT interval for ECG{LEAD}, measuring time between Q and T waves	ECG
20	ECG_{LEAD}_T-amplitude	T-wave amplitude for ECG{LEAD}, reflecting ventricular repolarization strength	ECG
21	BMI	Body Mass Index, representing body fat based on height and weight	
22	Age	Subject age	
23	Gender	Subject gender	
24	Race	Race as 1 hot encoding of white, asian, hispanic or african descent	

Supplementary Table 14 Hyperparameter description and values used for the creation of the random forest model

Hyperparameter	Value	Description
Number of Trees	{50, 100, 200, 300, 400, 500}	Specifies the number of decision trees in the forest.
Method of Modeling	Regression	The model predicts continuous target values (regression task).
Out-of-Bag Predictions	Enabled	Allows model validation using samples not used in training (out-of-bag samples).
Out-of-Bag Prediction Importance	Enabled	Identifies the importance of each feature in predicting the target.
Curvature Predictor Selection	Enabled	Measures the importance of each feature using curvature criteria.
MinLeafSize	**5**	Minimum number of leaf node observations

References

1 Breiman, L. Random forests. *Machine learning* **45**, 5-32 (2001).

2 Mukkamala, R., Stergiou, G. S. & Avolio, A. P. Cuffless blood pressure measurement. *Annual Review of Biomedical Engineering* **24**, 203-230 (2022).

3 Schutte, A. E., Kollias, A. & Stergiou, G. S. Blood pressure and its variability: classic and novel measurement techniques. *Nature Reviews Cardiology* **19**, 643-654 (2022).

4 Picone, D. S. *et al.* Accuracy of cuff-measured blood pressure: systematic reviews and meta-analyses. *Journal of the American College of Cardiology* **70**, 572-586 (2017).

5 Stergiou, G. S. *et al.* Antihypertensive treatment based on conventional or ambulatory blood pressure measurement: a randomized controlled trial. *Journal of hypertension* **39**, 1293-1302 (2021).

6 Stergiou, G. S. *et al.* Phenotypes of masked hypertension: isolated ambulatory, isolated home and dual masked hypertension. *Journal of hypertension* **38**, 218-223 (2020).

7 Mukkamala, R. *et al.* Evaluation of the accuracy of cuffless blood pressure measurement devices: challenges and proposals. *Hypertension* **78**, 1161-1167 (2021).

8 Association, I. S. & others. IEEE standard for wearable, cuffless blood pressure measuring devices–Amendment 1 (Amendment to IEEE Std 1708–2014). *IEEE Std. 1708a-2019* **19**, 1-35 (2019).

9 Armoundas, A. A. *et al.* Controversy in Hypertension: Pro-side of the Argument Using AI for Hypertension Diagnosis and Management. *HYPE*, doi:HYPE/2023/22349.R2 (2023).

10 Au-Yeung, W. M., Sahani, A. K., Isselbacher, E. M. & Armoundas, A. A. Reduction of false alarms in the intensive care unit using an optimized machine learning based approach. *NPJ digital medicine* **2**, 86, doi:10.1038/s41746-019-0160-7 (2019).

11 Radha, M. *et al.* Arterial path selection to measure pulse wave velocity as a surrogate marker of blood pressure. *Biomedical Physics & Engineering Express* **3** (2017).

12 Ding, X. *et al.* Pulse transit time based continuous cuffless blood pressure estimation: A new extension and a comprehensive evaluation. *Scientific reports* **7**, 1-11 (2017).

13 Slapničar, G., N, M. & Luštrek, M. Blood pressure estimation from photoplethysmogram using a spectro-temporal deep neural network. *Sensors* **19** (2019).

14 Simjanoska, M., Gjoreski, M., M, G. & Madevska Bogdanova, A. Non-invasive blood pressure estimation from ECG using machine learning techniques. *Sensors* **18** (2018).

15 Kachuee, M., Kiani, M. M., H, M. & Shabany, M. 1006-1009.

16 Wang, L., Zhou, W., Y, X. & Zhou, X. A novel neural network model for blood pressure estimation using photoplethesmography without electrocardiogram. *Journal of healthcare engineering* **2018** (2018).

17 O'Brien, E., Waeber, B., Parati, G., Staessen, J. & Myers, M. G. Blood pressure measuring devices: recommendations of the European Society of Hypertension. *Bmj* **322**, 531-536 (2001).

Supplementary Figure 1 - Box-plots of feature importance scores. Distribution of feature importance scores of the five RF models (one for each fold during cross-validation), in the order presented in Supplementary Table 3, for systolic blood pressure (BP) estimation.

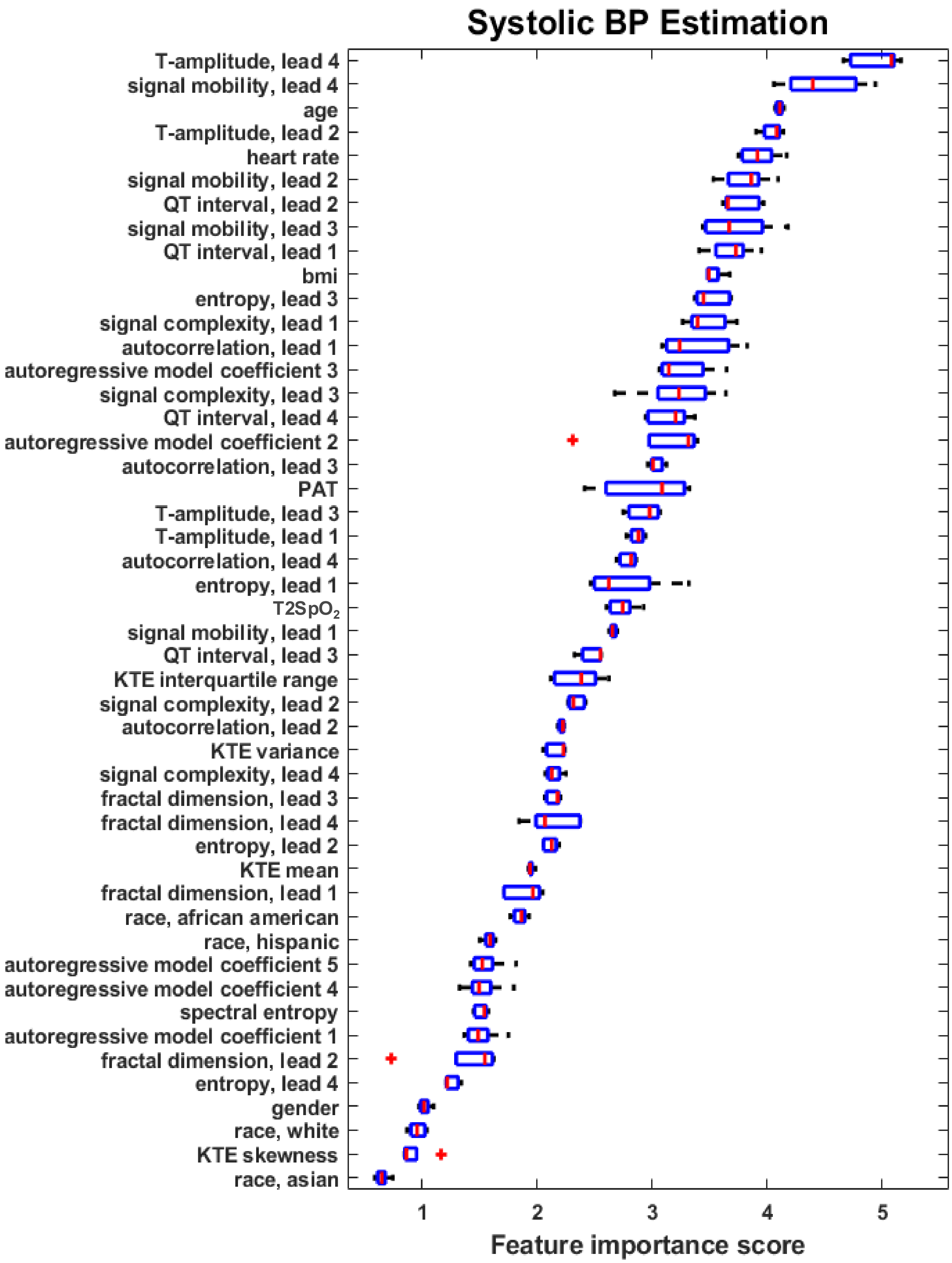

Supplementary Figure 2 - Box-plots of feature importance scores. Distribution of feature importance scores of the five RF models (one for each fold during cross-validation), in the order presented in Supplementary Table 3, for diastolic blood pressure (BP) estimation.

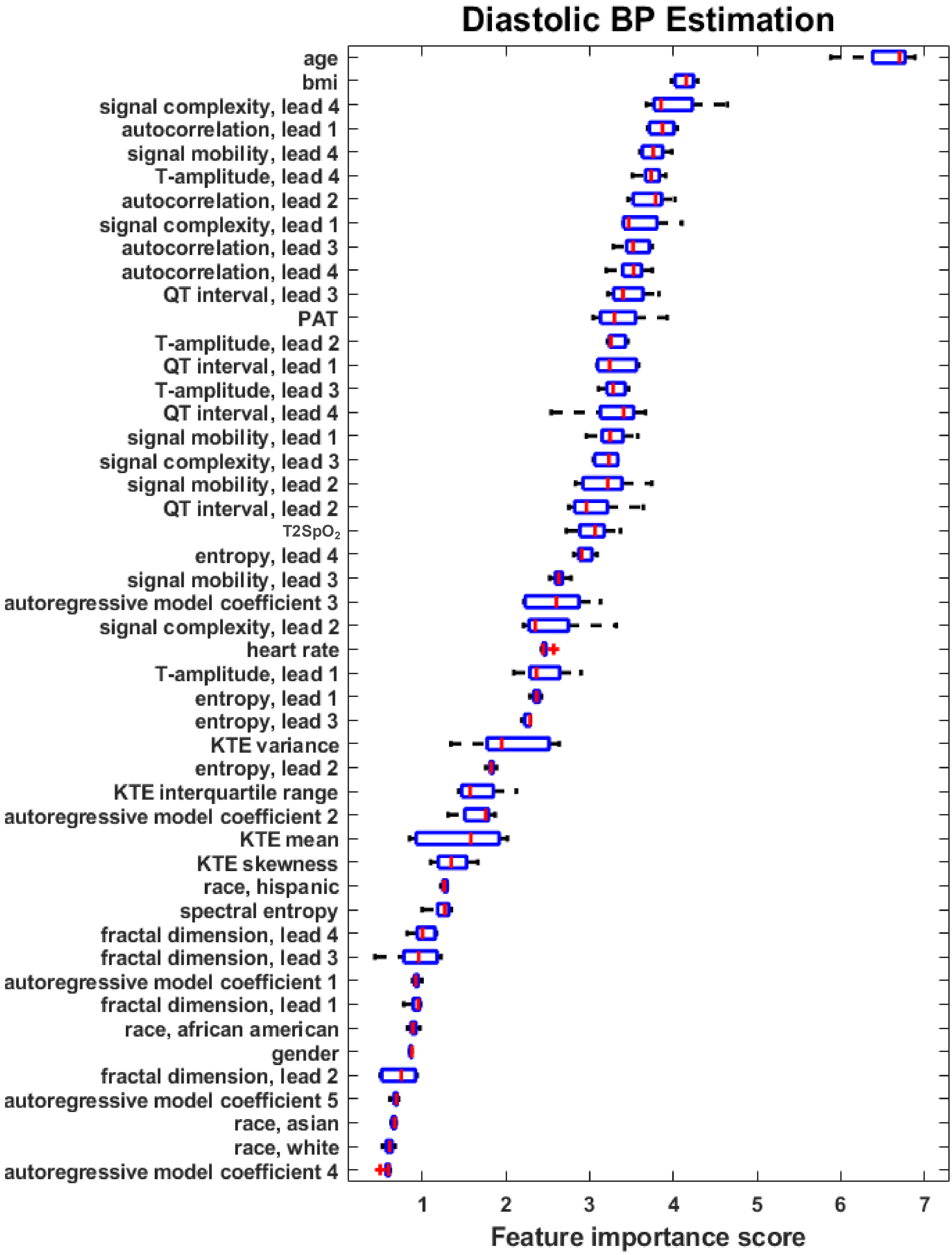

Supplementary Figure 3 - Absolute error estimation across age groups for systolic blood pressure estimation. The number of employed sequences pertaining to each box-plot range are mentioned at the top of the figure, with the mean absolute error shown below, along with the average ± standard deviation of the group demographic characteristics: age (in years), gender (1 for female, 2 for male), race (0 for Asian, 1 for Black, 2 for Hispanic, 3 for White), BMI (in kg/m^2).. The blue box spans the interquartile range (IQR, 75%-25%), covering the median (50%, red line) of the data from the first quartile (Q1, 25th percentile) to the third quartile (Q3, 75th percentile). The black caps (upper and lower horizontal lines extend to ± 1.5 times IQR) indicate the furthest data points that are still within the expected range before classifying values as outliers. Red markers indicate outliers, representing extreme errors. A linear mixed model (LMM) was employed to account for multiple samples from the same subject, within each age group. The results (p = 0.79) indicate no statistically significant influence of age on blood pressure absolute error.

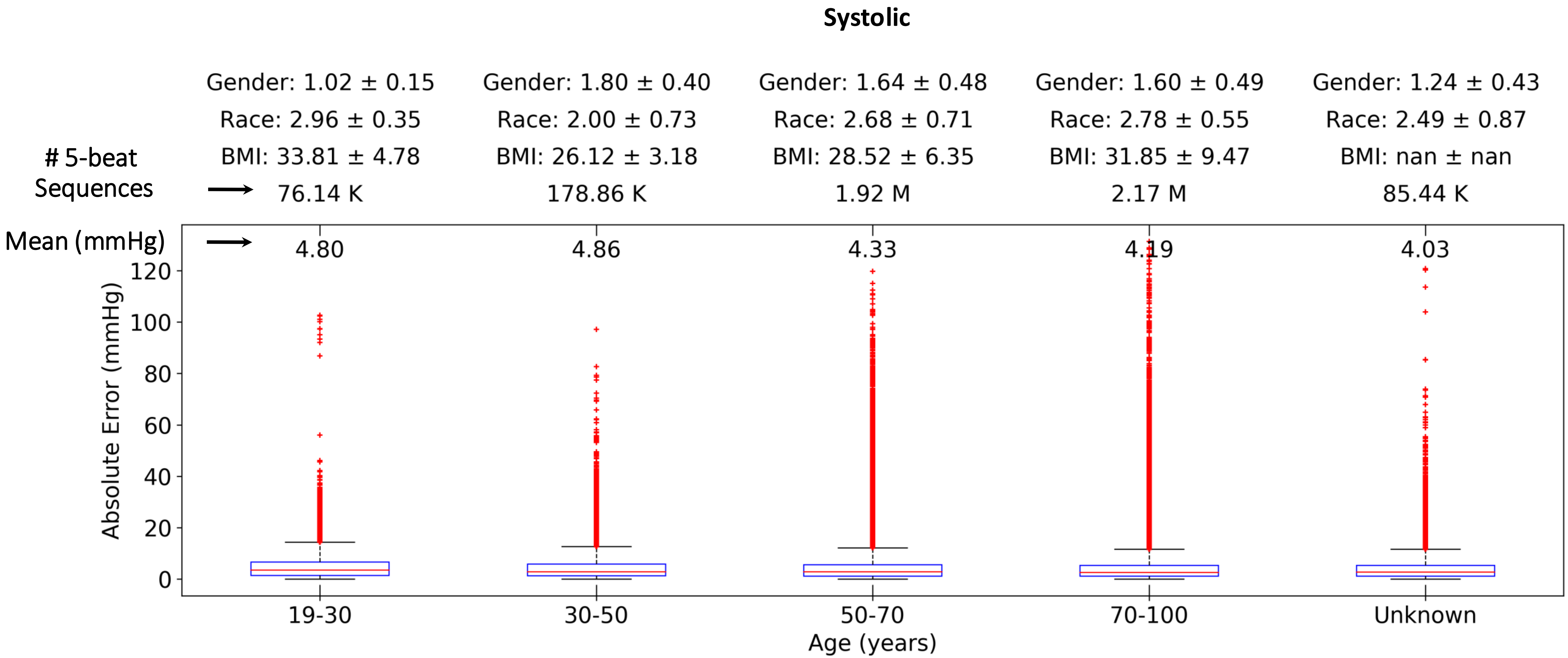

Supplementary Figure 4 Absolute error estimation across age groups for diastolic blood pressure (BP) estimation. The number of employed sequences pertaining to each box-plot range are mentioned at the top of the figure, with the mean absolute error shown below, along with the average ± standard deviation of the group demographic characteristics: age (in years), gender (1 for female, 2 for male), race (0 for Asian, 1 for Black, 2 for Hispanic, 3 for White), BMI (in kg/m^2).. The blue box spans the interquartile range (IQR, 75%-25%), covering the median (50%, red line) of the data from the first quartile (Q1, 25th percentile) to the third quartile (Q3, 75th percentile). The black caps (upper and lower horizontal lines extend to ± 1.5 times IQR) indicate the furthest data points that are still within the expected range before classifying values as outliers. Red markers indicate outliers, representing extreme errors. A linear mixed model (LMM) was employed to account for multiple samples from the same subject, within each age group. The results (p = 0.73) indicate no statistically significant influence of age on BP absolute error.

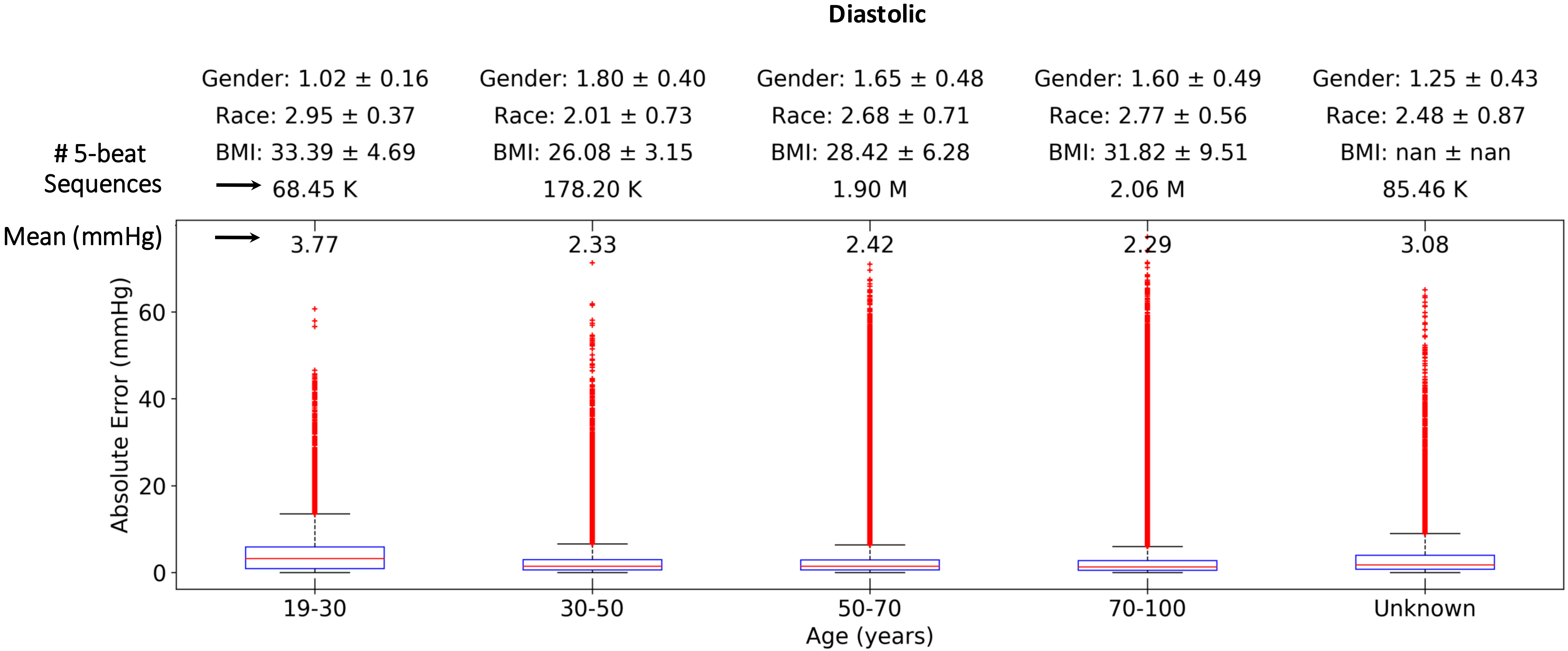

Supplementary Figure 5 - Absolute error estimation across gender groups for systolic blood pressure (BP) estimation. The number of employed sequences pertaining to each box-plot range are mentioned at the top of the figure, with the mean absolute error shown below, along with the average ± standard deviation of the group demographic characteristics: age (in years), gender (1 for female, 2 for male), race (0 for Asian, 1 for Black, 2 for Hispanic, 3 for White), BMI (in kg/m^2).. The blue box spans the interquartile range (IQR, 75%-25%), covering the median (50%, red line) of the data from the first quartile (Q1, 25th percentile) to the third quartile (Q3, 75th percentile). The black caps (upper and lower horizontal lines extend to ± 1.5 times IQR) indicate the furthest data points that are still within the expected range before classifying values as outliers. Red markers indicate outliers, representing extreme errors. A linear mixed model (LMM) was employed to account for multiple samples from the same subject, within each gender group. The results (p = 0.27) indicate no statistically significant influence of gender on BP absolute error.

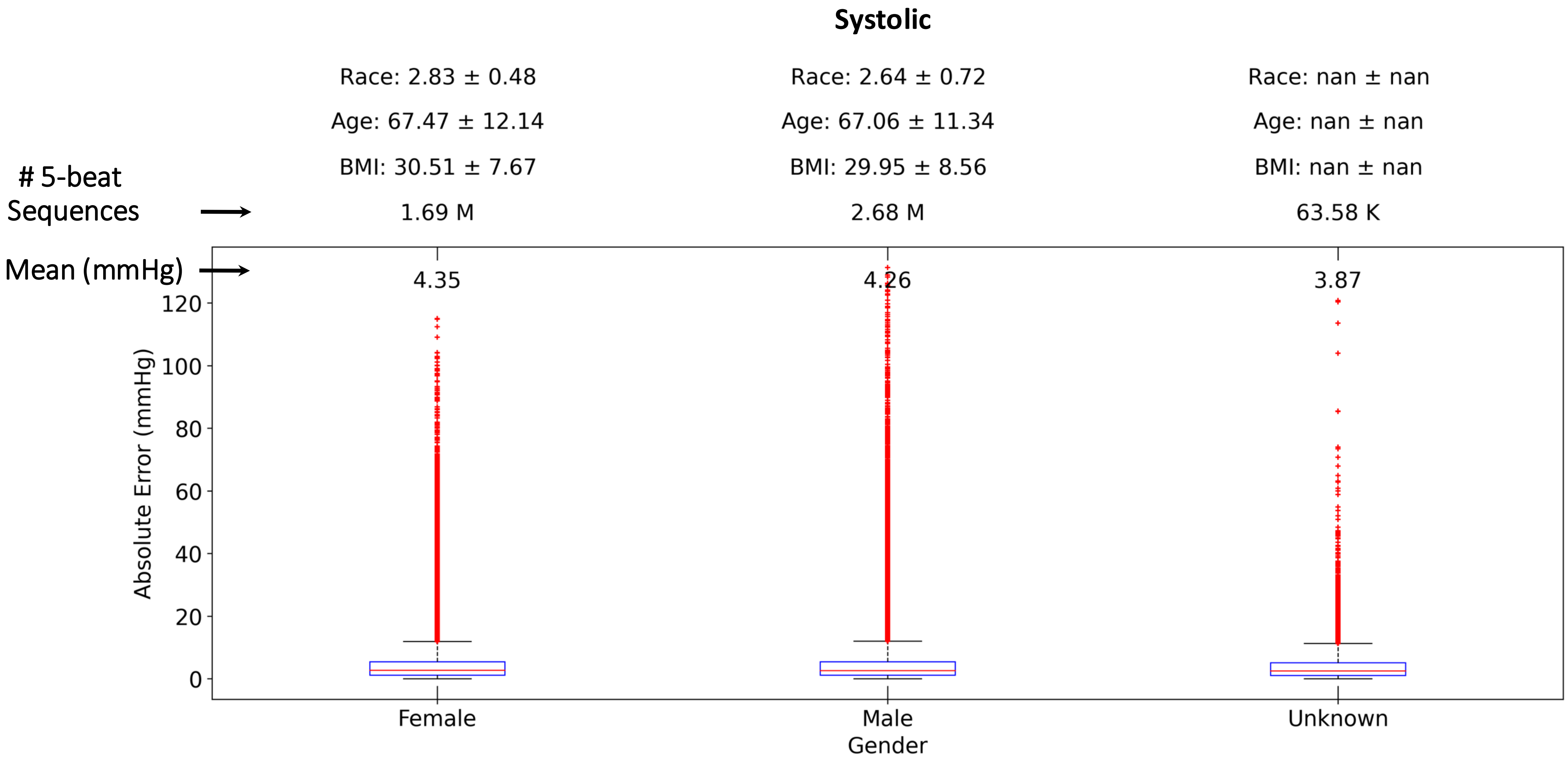

Supplementary Figure 6 - Absolute error estimation across gender groups for diastolic blood pressure (BP) estimation. The number of employed sequences pertaining to each box-plot range are mentioned at the top of the figure, with the mean absolute error shown below, along with the average ± standard deviation of the group demographic characteristics: age (in years), gender (1 for female, 2 for male), race (0 for Asian, 1 for Black, 2 for Hispanic, 3 for White), BMI (in kg/m^2).. The blue box spans the interquartile range (IQR, 75%-25%), covering the median (50%, red line) of the data from the first quartile (Q1, 25th percentile) to the third quartile (Q3, 75th percentile). The black caps (upper and lower horizontal lines extend to ± 1.5 times IQR) indicate the furthest data points that are still within the expected range before classifying values as outliers. Red markers indicate outliers, representing extreme errors. A linear mixed model (LMM) was employed to account for multiple samples from the same subject, within each gender group. The results (p = 0.09) indicate no statistically significant influence of gender on BP absolute error.

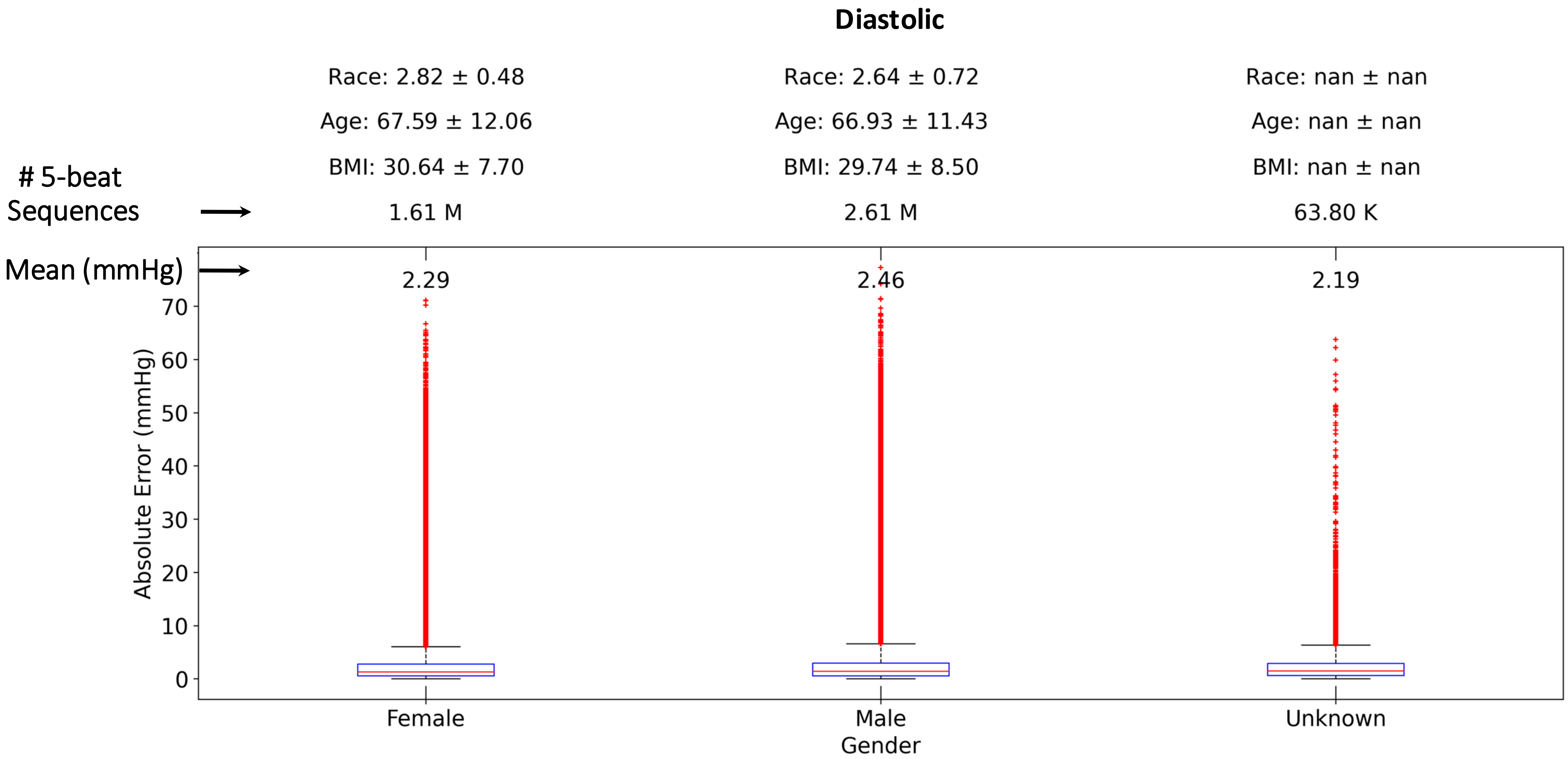

Supplementary Figure 7 - Absolute error estimation across race groups for systolic blood pressure (BP) estimation. The number of employed sequences pertaining to each box-plot range are mentioned at the top of the figure, with the mean absolute error shown below, along with the average ± standard deviation of the group demographic characteristics: age (in years), gender (1 for female, 2 for male), race (0 for Asian, 1 for Black, 2 for Hispanic, 3 for White), BMI (in kg/m^2).. The blue box spans the interquartile range (IQR, 75%-25%), covering the median (50%, red line) of the data from the first quartile (Q1, 25th percentile) to the third quartile (Q3, 75th percentile). The black caps (upper and lower horizontal lines extend to ± 1.5 times IQR) indicate the furthest data points that are still within the expected range before classifying values as outliers. Red markers indicate outliers, representing extreme errors. A linear mixed model (LMM) was employed to account for multiple samples from the same subject, within each race group. The results (p = 0.22) indicate no statistically significant influence of race on BP absolute error.

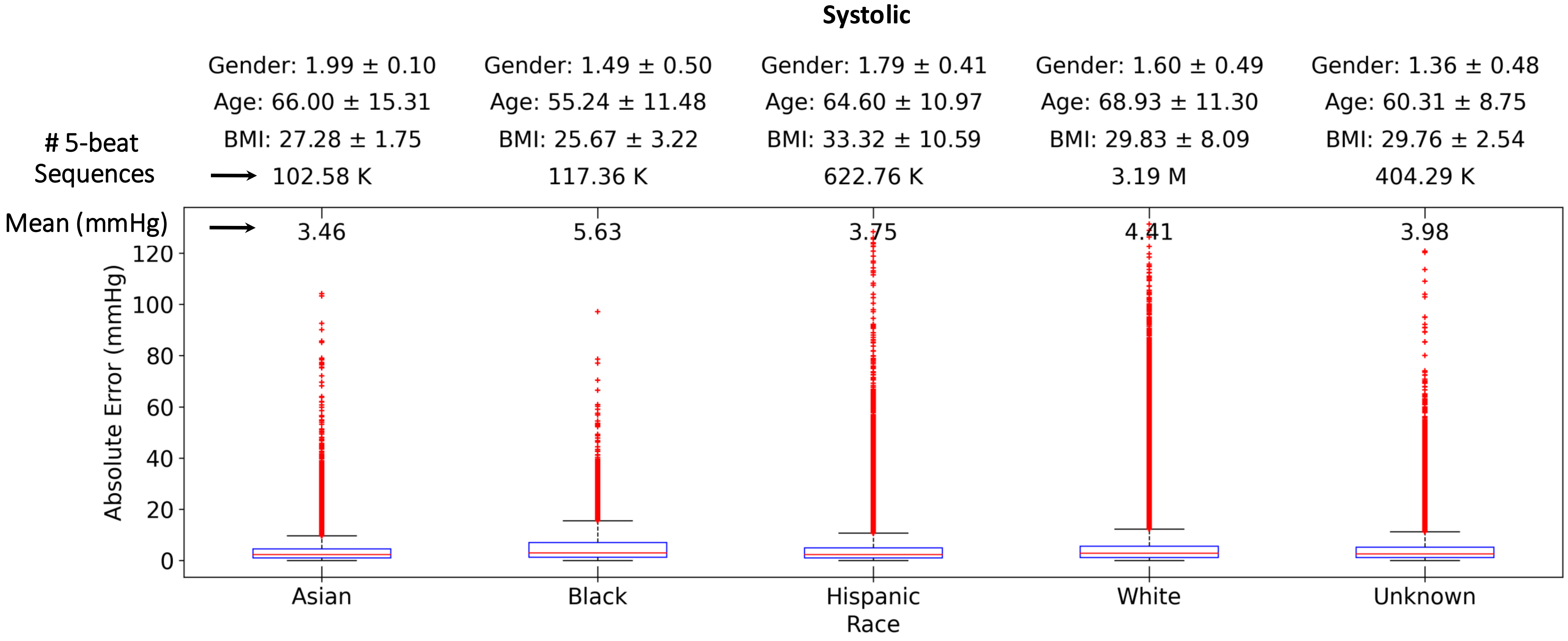

Supplementary Figure 8 - Absolute error estimation across race groups for diastolic blood pressure (BP) estimation. The number of employed sequences pertaining to each box-plot range are mentioned at the top of the figure, with the mean absolute error shown below, along with the average ± standard deviation of the group demographic characteristics: age (in years), gender (1 for female, 2 for male), race (0 for Asian, 1 for Black, 2 for Hispanic, 3 for White), BMI (in kg/m^2).. The blue box spans the interquartile range (IQR, 75%-25%), covering the median (50%, red line) of the data from the first quartile (Q1, 25th percentile) to the third quartile (Q3, 75th percentile). The black caps (upper and lower horizontal lines extend to ± 1.5 times IQR) indicate the furthest data points that are still within the expected range before classifying values as outliers. Red markers indicate outliers, representing extreme errors. A linear mixed model (LMM) was employed to account for multiple samples from the same subject, within each race group. The results (p = 0.29) indicate no statistically significant influence of race on BP absolute error.

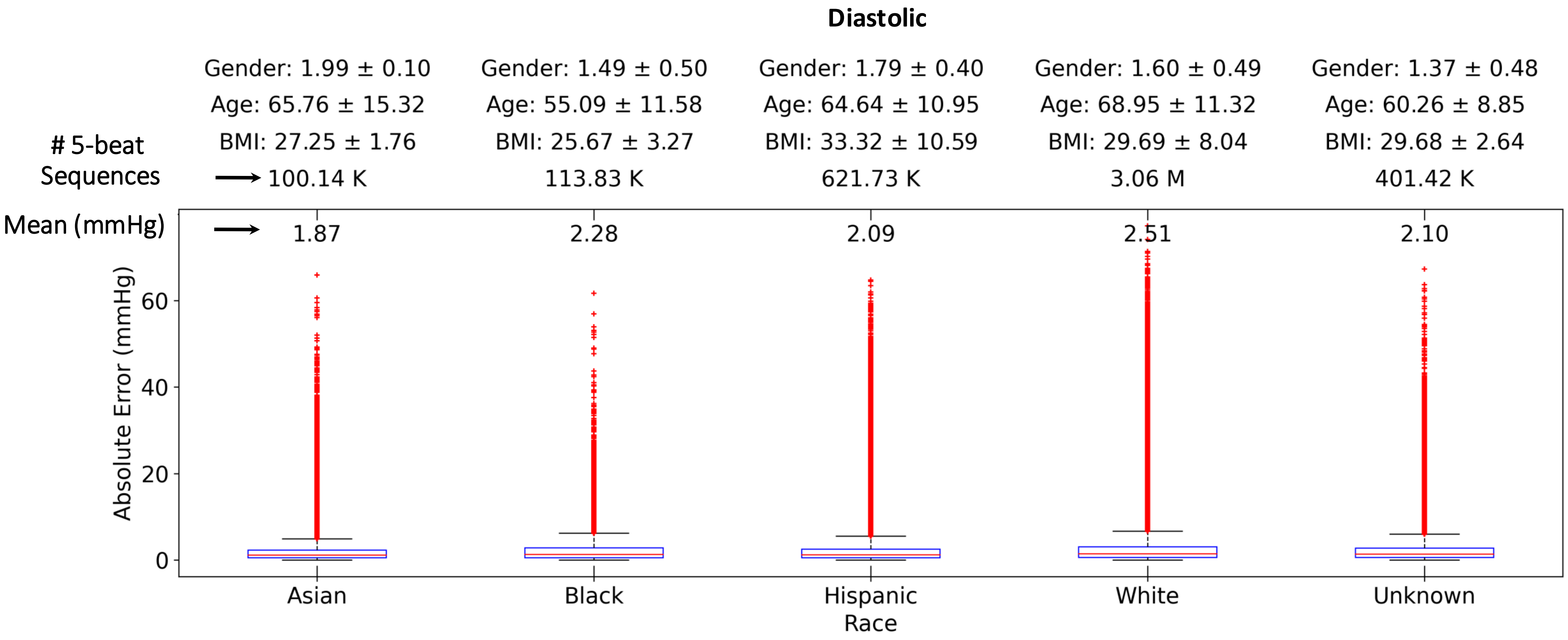

Supplementary Figure 9 - Estimation of the absolute error of systolic blood pressure values, across each fold. The number of employed sequences pertaining to each box-plot range, are mentioned at the top of the figure, with the mean absolute error shown below. Box plots indicate the blood pressure (BP) absolute error distribution across the different folds. The blue box spans the interquartile range (IQR, 75%-25%), covering the median (50%, red line) of the data from the first quartile (Q1, 25th percentile) to the third quartile (Q3, 75th percentile). The black caps (upper and lower horizontal lines extend to ± 1.5 times IQR) indicate the furthest data points that are still within the expected range before classifying values as outliers. Red markers indicate outliers, representing extreme errors. A LMM model was employed to account for multiple samples from the same subject, within each and across folds. The results indicate that BP absolute error does not significantly differ across the different folds ($p = 0.53$).

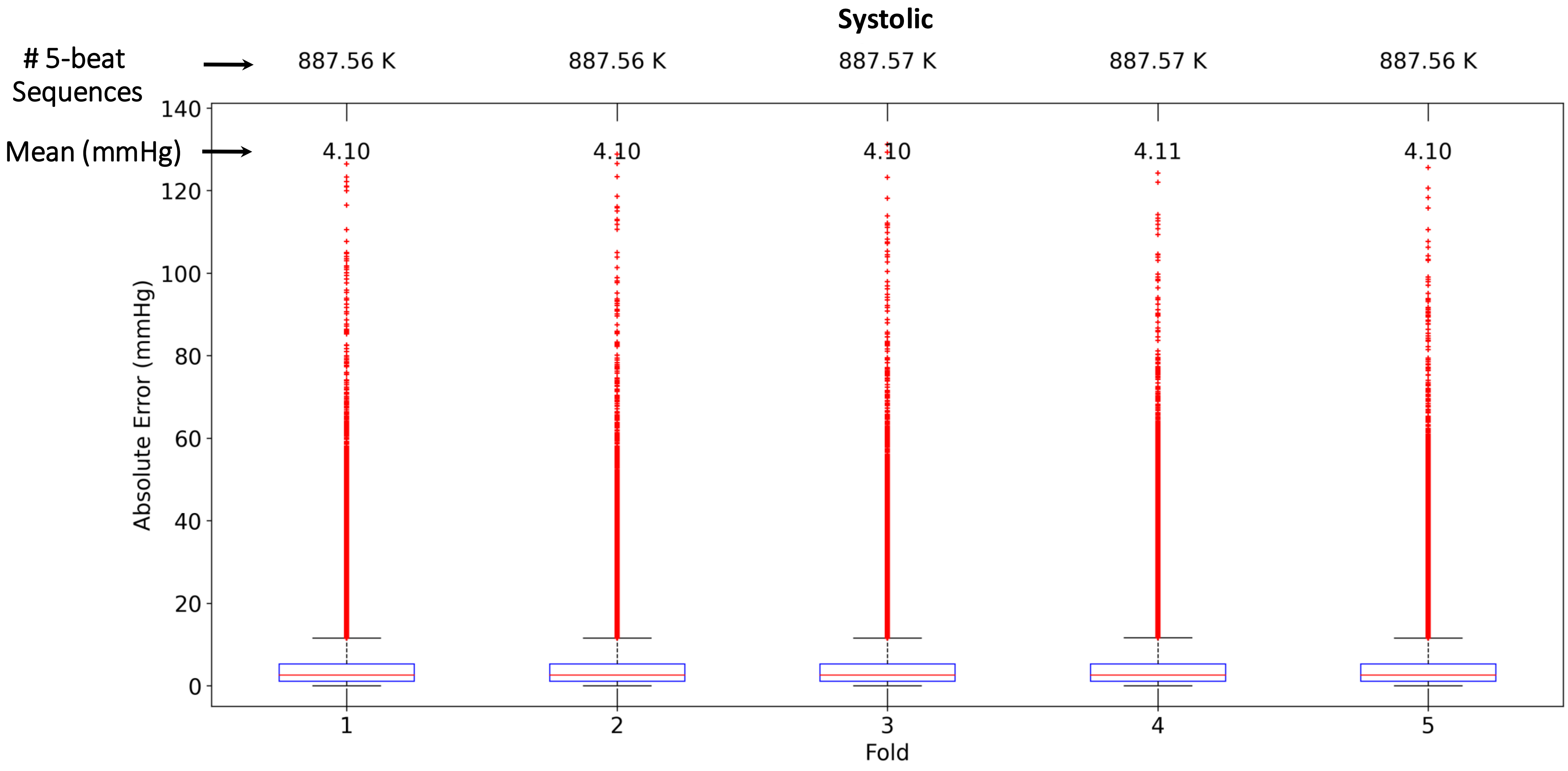

Supplementary Figure 10 - Estimation of the absolute error of diastolic blood pressure values, across each fold. The number of employed sequences pertaining to each box-plot range, are mentioned at the top of the figure, with the mean absolute error shown below. Box plots indicate the blood pressure (BP) absolute error distribution across the different folds. The blue box spans the interquartile range (IQR, 75%-25%), covering the median (50%, red line) of the data from the first quartile (Q1, 25th percentile) to the third quartile (Q3, 75th percentile). The black caps (upper and lower horizontal lines extend to ± 1.5 times IQR) indicate the furthest data points that are still within the expected range before classifying values as outliers. Red markers indicate outliers, representing extreme errors. A LMM model was employed to account for multiple samples from the same subject, within each and across folds. The results indicate that BP absolute error does not significantly differ across the different folds ($p = 0.88$).

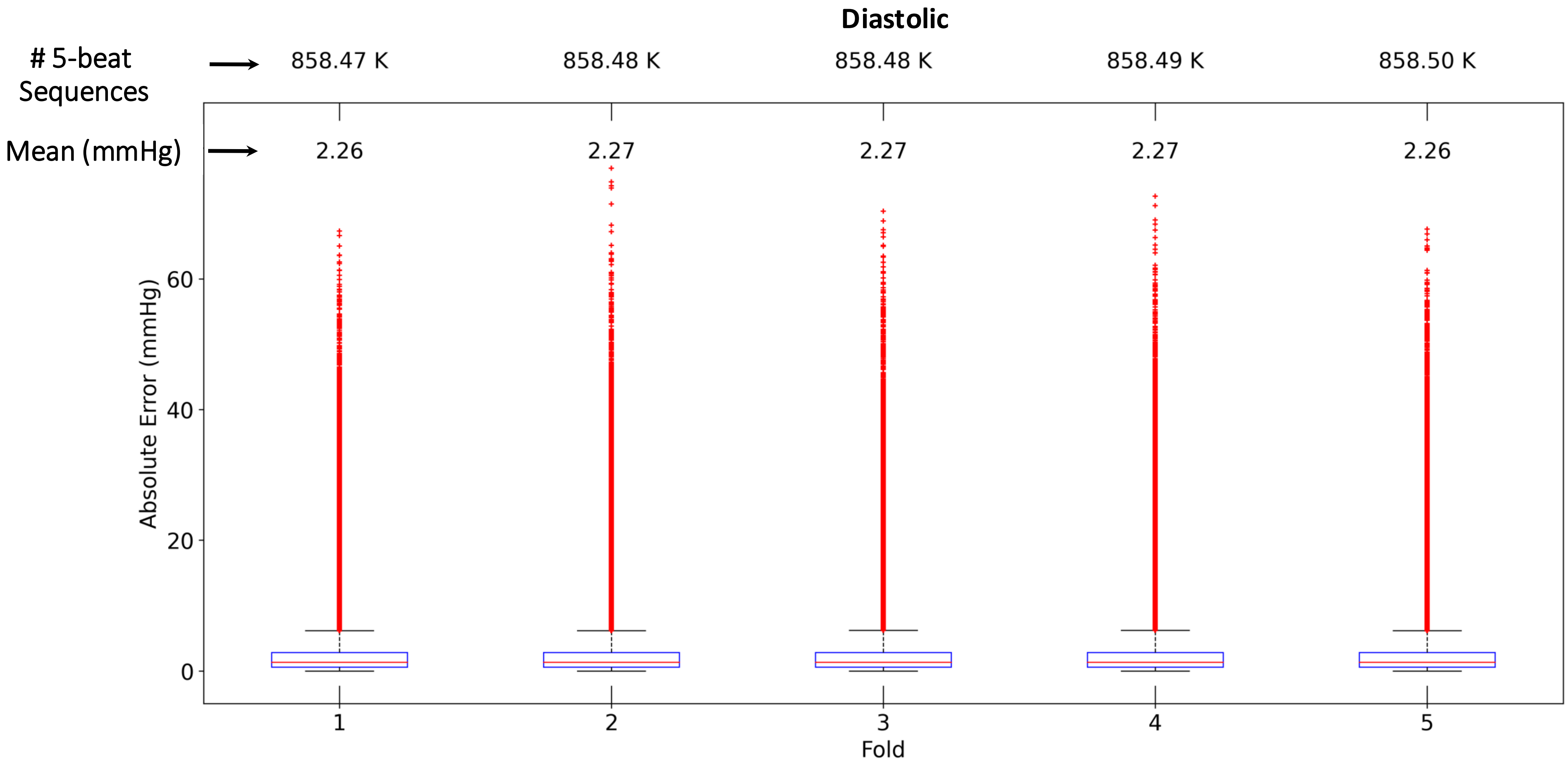

Supplementary Figure 11 - Example of the correlation between the systolic estimated and true blood pressure values using the augmented subject-specific model, across all subjects. The red dashed line is the identity line, indicating perfect agreement between estimated and true values. The correlation coefficient (R=0.95) indicates the highly linear relationship between the estimated and the true values (p<10-6).

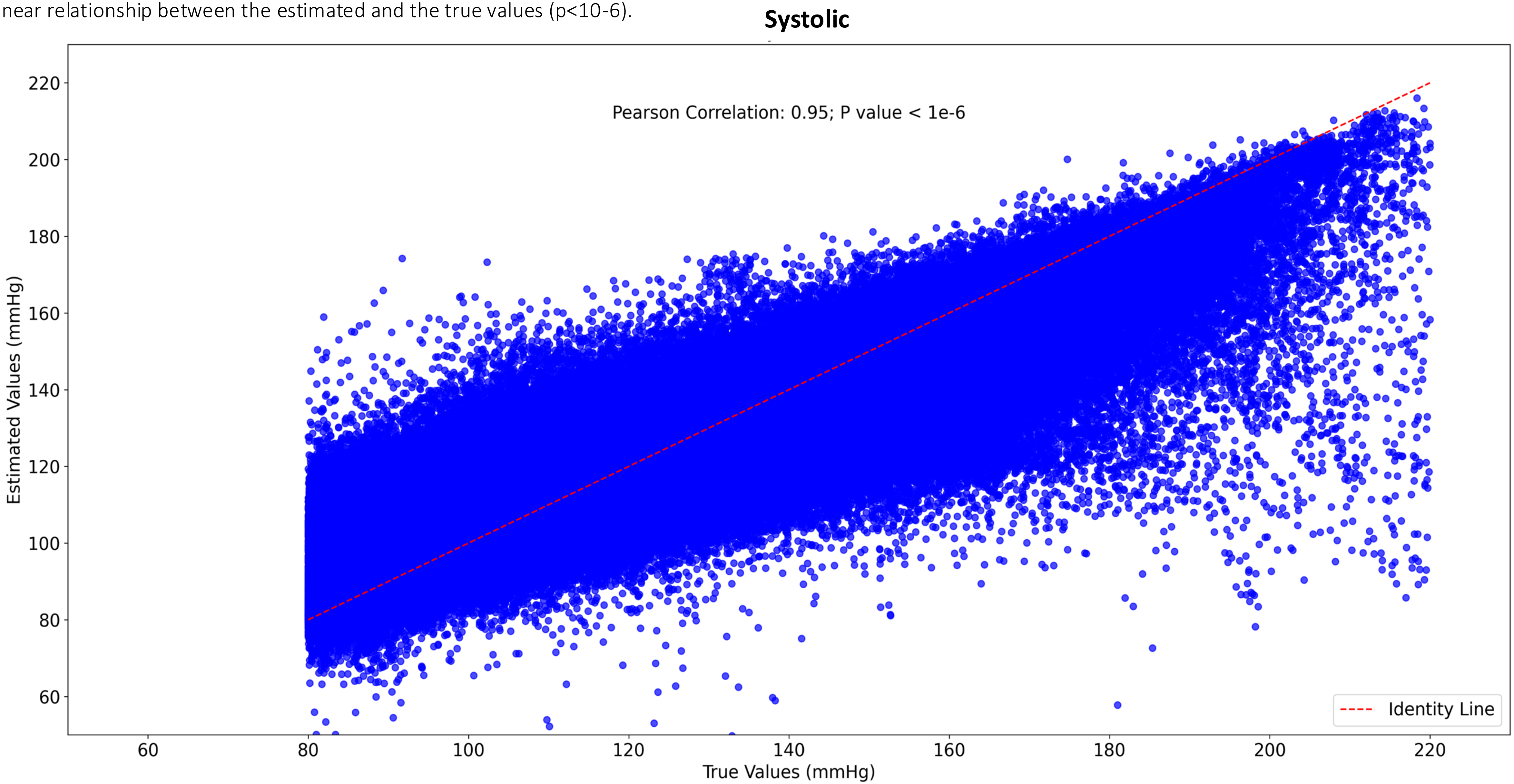

Supplementary Figure 12 - Example of the correlation between the diastolic estimated and true blood pressure values using the augmented subject-specific model, across all subjects. The red dashed line is the identity line, indicating perfect agreement between estimated and true values. The correlation coefficient (R=0.92) indicates the highly linear relationship between the estimated and the true values (p<10-6).

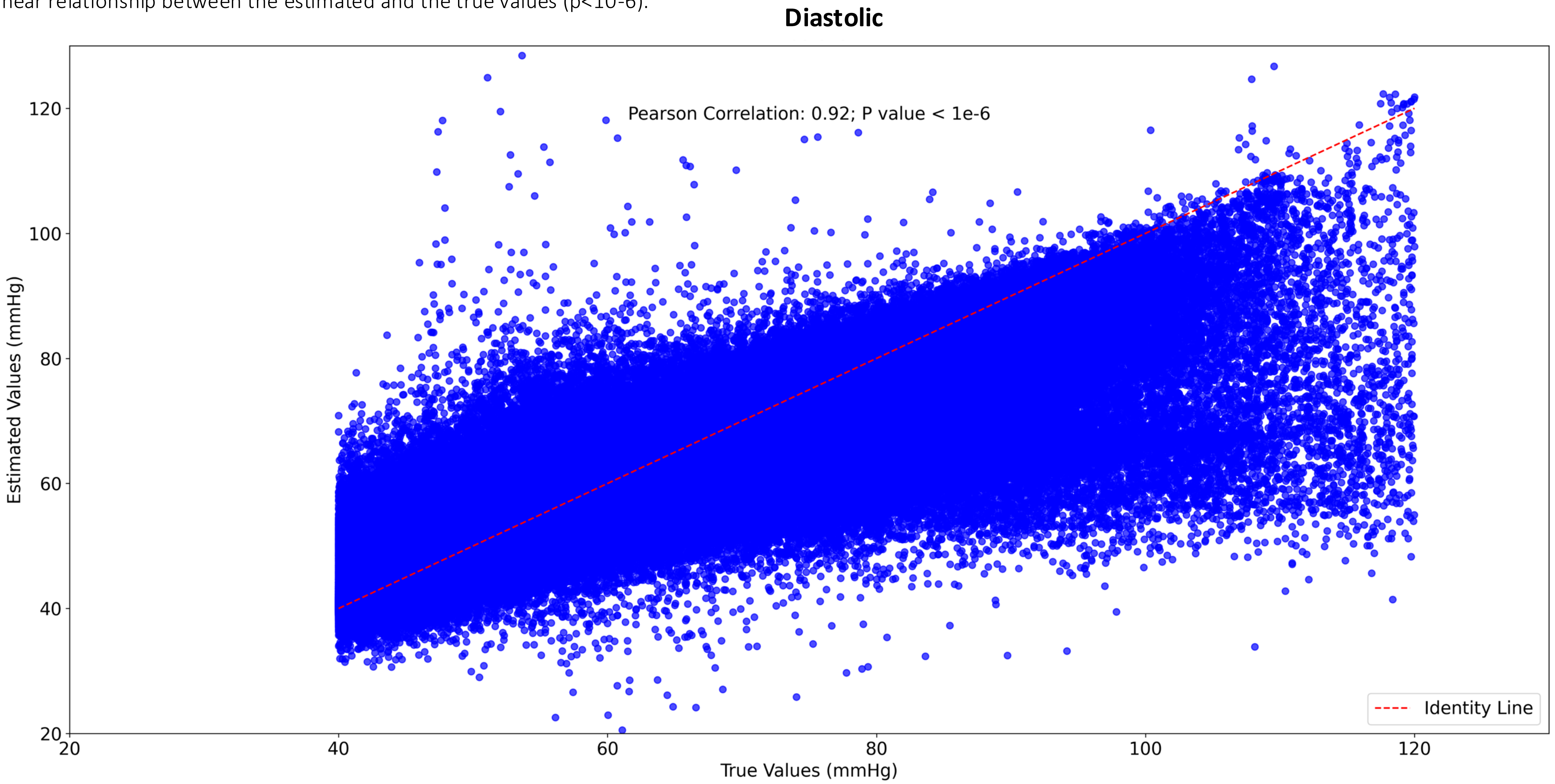

Supplementary Figure 13 - Bland-Altman plot of the systolic blood pressure demonstrating the agreement between the estimated and the true values. Plot of the estimated minus the true blood pressure (BP) values (y-axis), against the mean BP values (x-axis), across all subjects. The red dashed line represents the mean difference (bias) between the two, while the blue dashed lines indicate the limits of agreement (LoA) within which 95 % of the differences between the methods fall, defined as the mean ± 1.96 standard deviations.

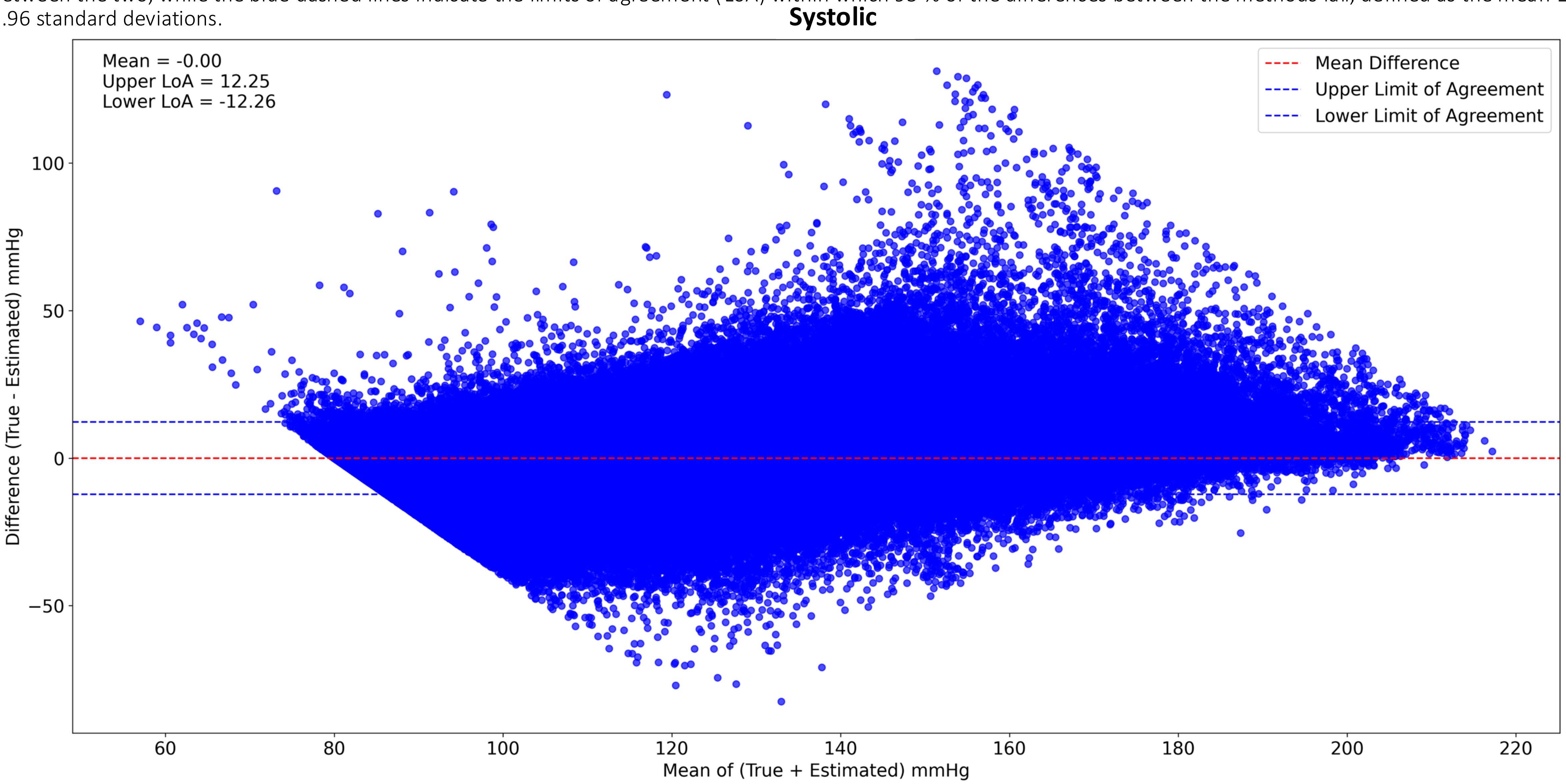

Supplementary Figure 14 - Bland-Altman plot of the diastolic blood pressure demonstrating the agreement between the estimated and the true values. Plot of the estimated minus the true blood pressure (BP) values (y-axis), against the mean BP values (x-axis), across all subjects. The red dashed line represents the mean difference (bias) between the two, while the blue dashed lines indicate the limits of agreement (LoA) within which 95 % of the differences between the methods fall, defined as the mean ± 1.96 standard deviations.

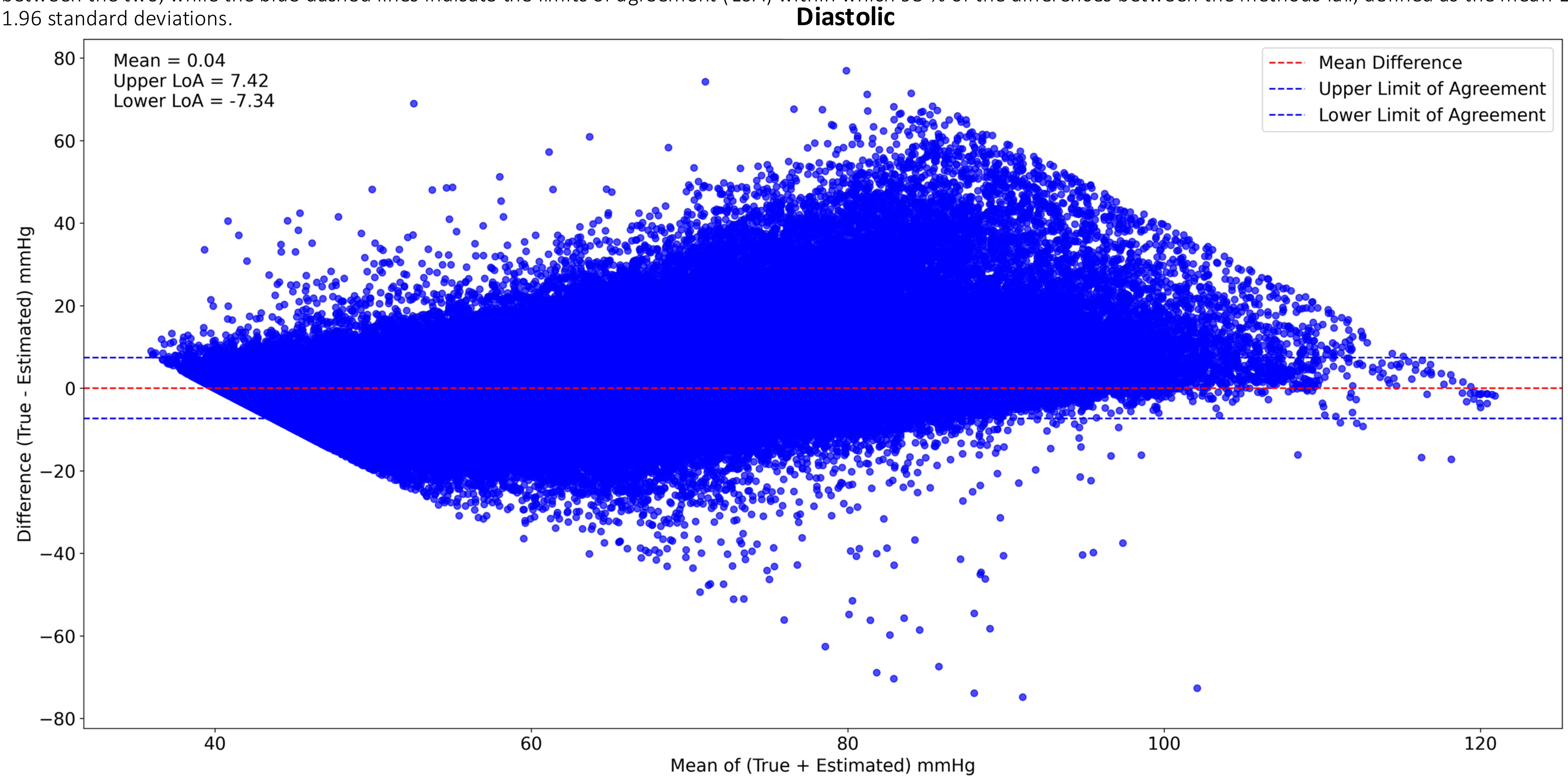

Supplementary Figure 15 - Comparison between systolic blood pressure estimation using the augmented patient-specific model (green) and the ground-truth reference (blue). The augmented model can effectively track the dynamic blood pressure (BP) amplitude fluctuations, demonstrating the proposed model's reliability in capturing gradual and abrupt changes. Additionally, a 20-sample windowed cross-correlation analysis was performed, which showed that the maximum correlation occurs at a time lag of zero samples, indicating that the model's predictions are aligned with the reference arterial BP values, validating its time responsiveness. A 20-sample window was used to capture local shifts in BP amplitude without being overshadowed by long-term trends, while also mitigating the effects of non-stationarity by ensuring that correlations are computed within a stable, short-term segment of the signal.

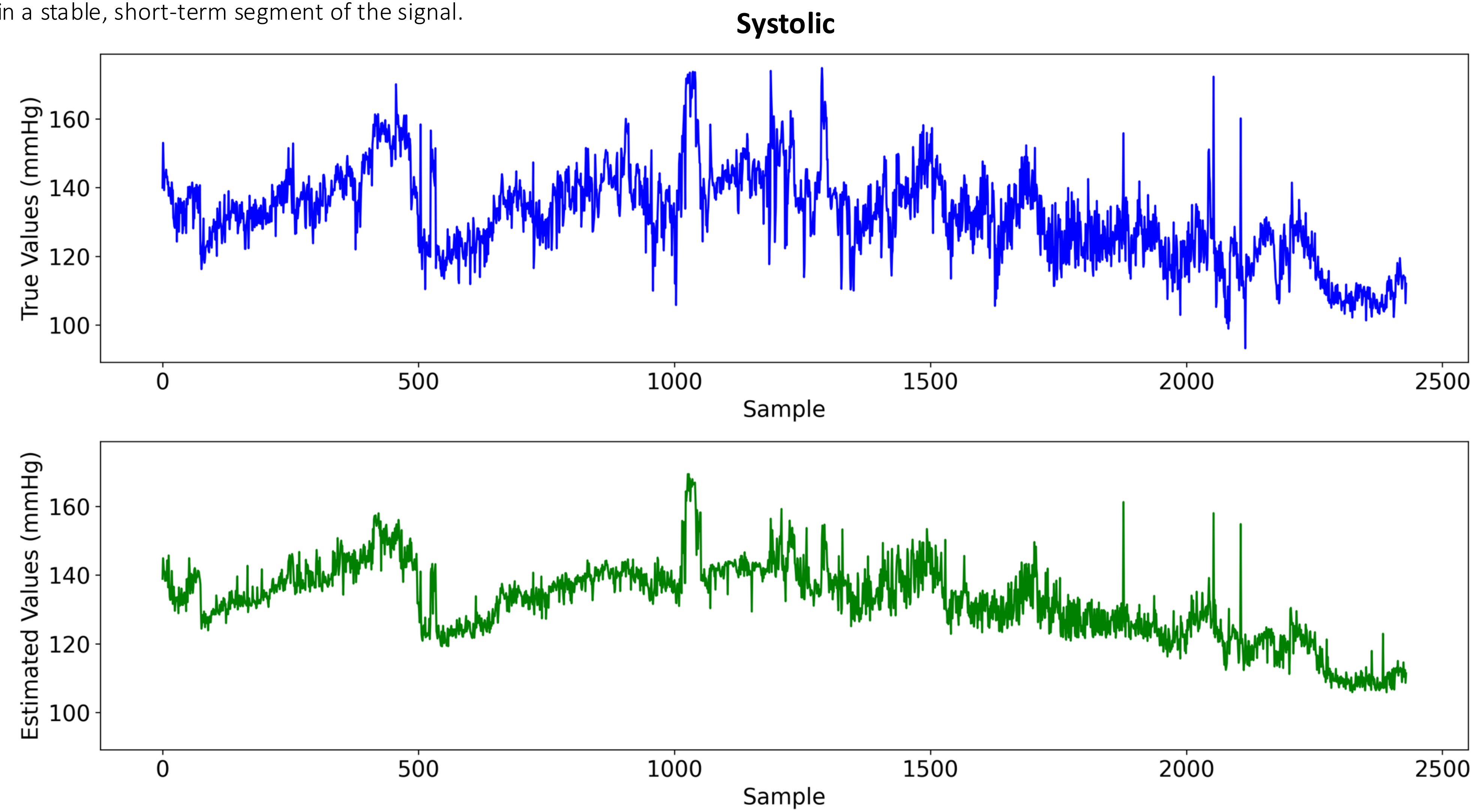

Supplementary Figure 16 - Comparison between diastolic blood pressure estimation using the augmented patient-specific model (green) and the ground-truth reference (blue). The augmented model can effectively track the dynamic blood pressure (BP) amplitude fluctuations, demonstrating the proposed model's reliability in capturing gradual and abrupt changes. Additionally, a 20-sample windowed cross-correlation analysis was performed, which showed that the maximum correlation occurs at a time lag of zero samples, indicating that the model's predictions are aligned with the reference arterial BP values, validating its time responsiveness. A 20-sample window was used to capture local shifts in BP amplitude without being overshadowed by long-term trends, while also mitigating the effects of non-stationarity by ensuring that correlations are computed within a stable, short-term segment of the signal.

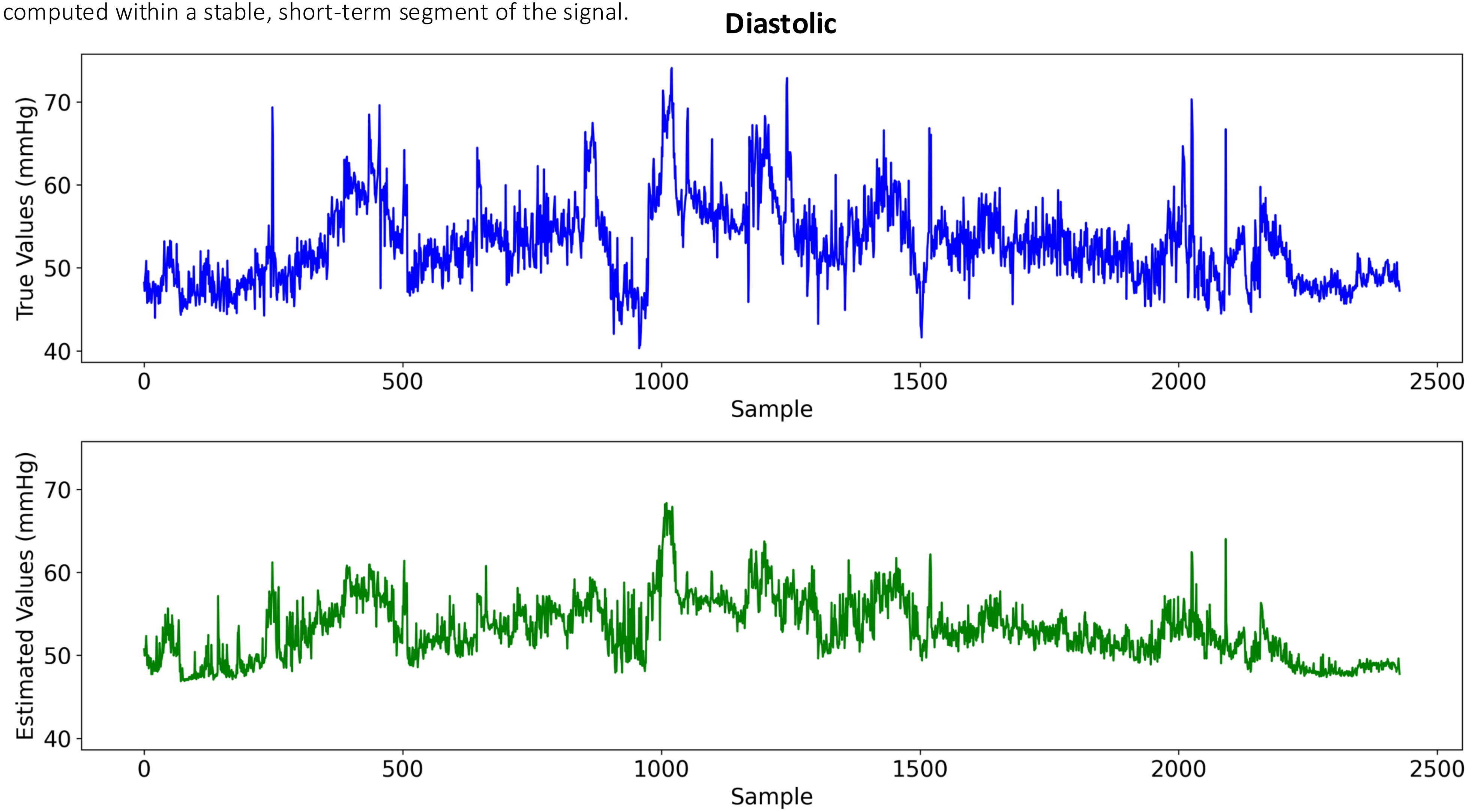

Supplementary Figure 17 - Hybrid-CNN architecture for noise detection in ECG. Given a 5-sec ECG signal/segment, our method uses signal processing techniques to extract hand-engineered features, and also uses convolutional layers to learn and extract additional features in determining noisy ECG s ignals. The fully connected layer at the end, acts as a classifier to discriminate the noisy from the clean ECG. The network weights of the convolutional layers and the fu lly connected layer, are learned from the annotated ECG segments during training phase, and they are frozen for use in test phase.

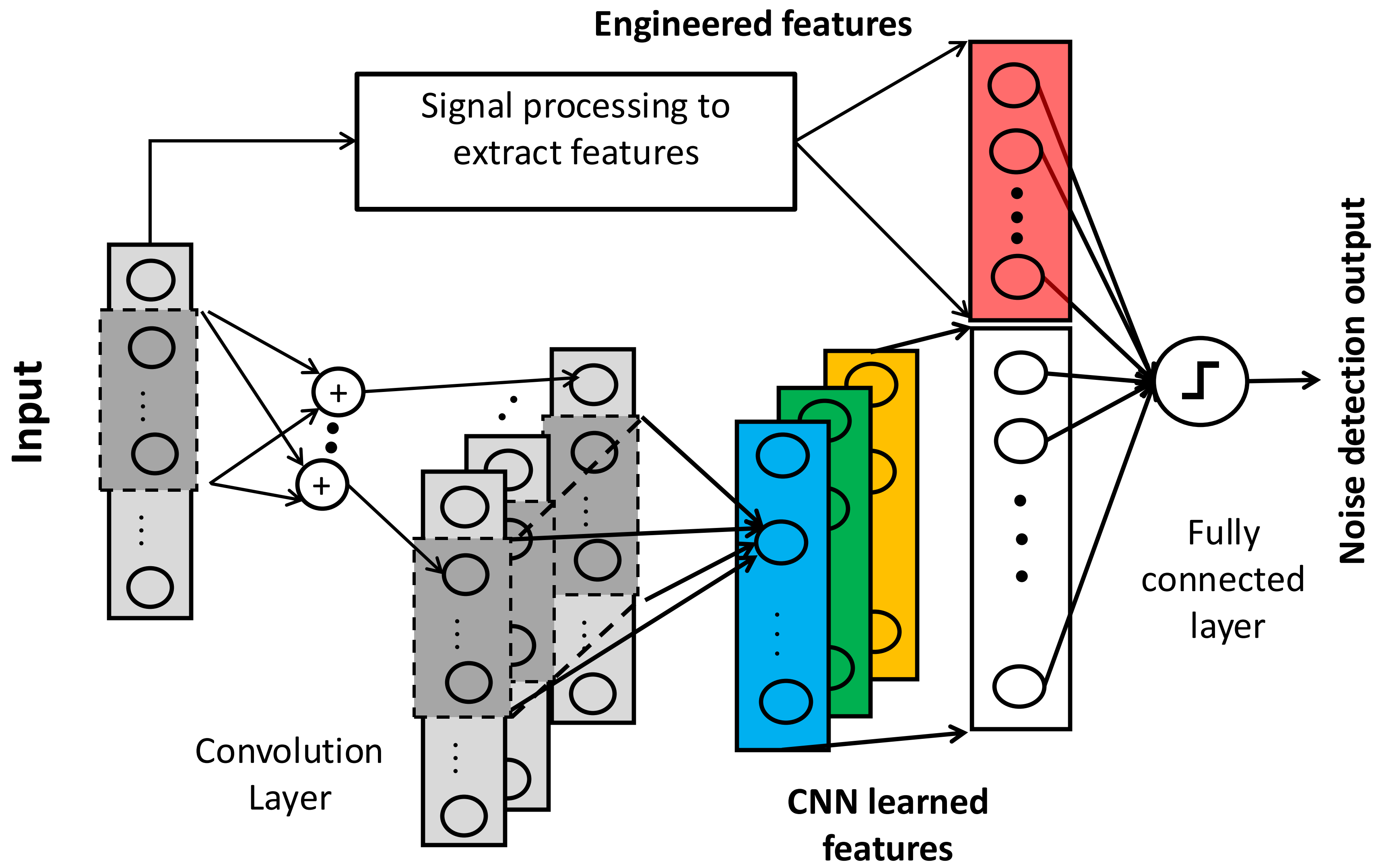

Supplementary Figure 18 - Representative signals annotated by 3 clinician experts as noisy, and non-noisy ECG signals, used for the training of the noise detection algorithm.

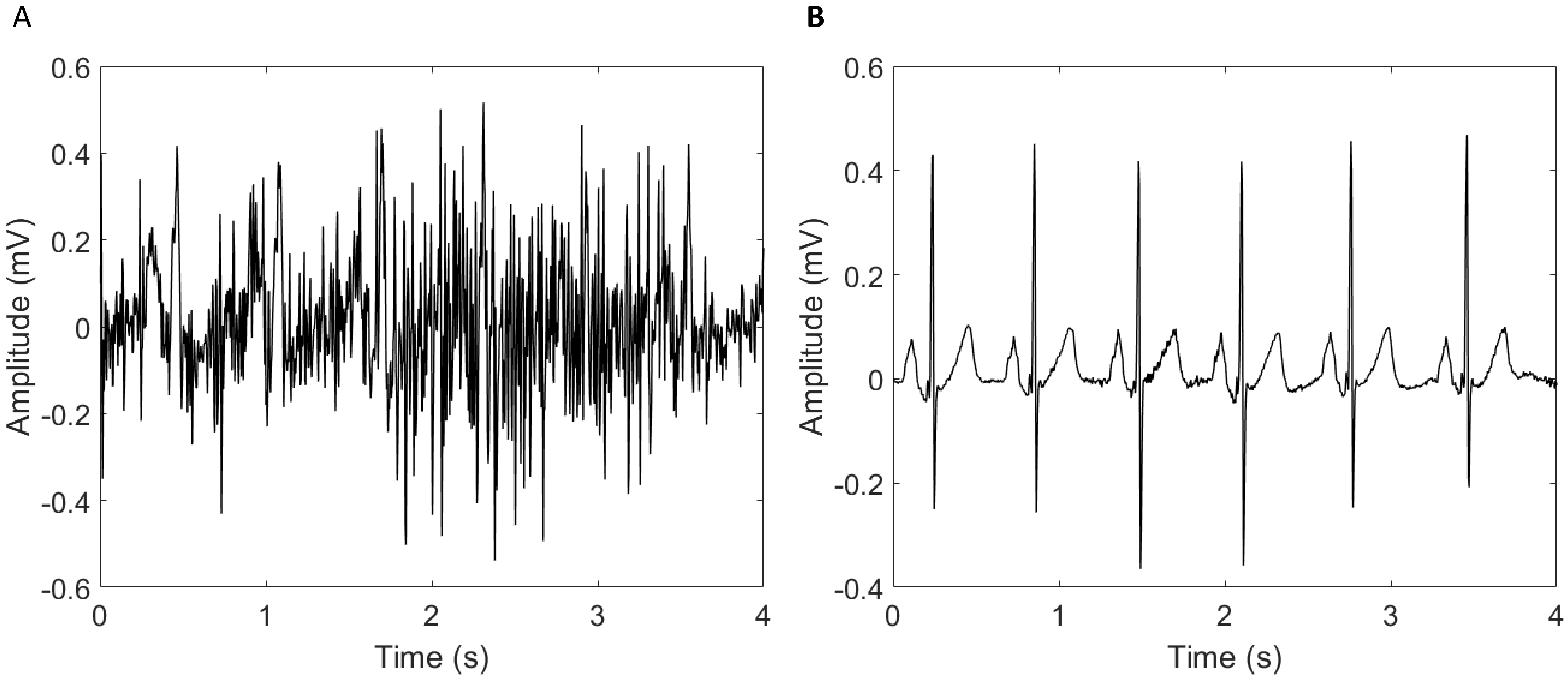

Supplementary Figure 19 - ECG raw and filtered signal across the four ECG leads before and after the application of a 51st order FIR filter with a 15Hz cut-off frequency.

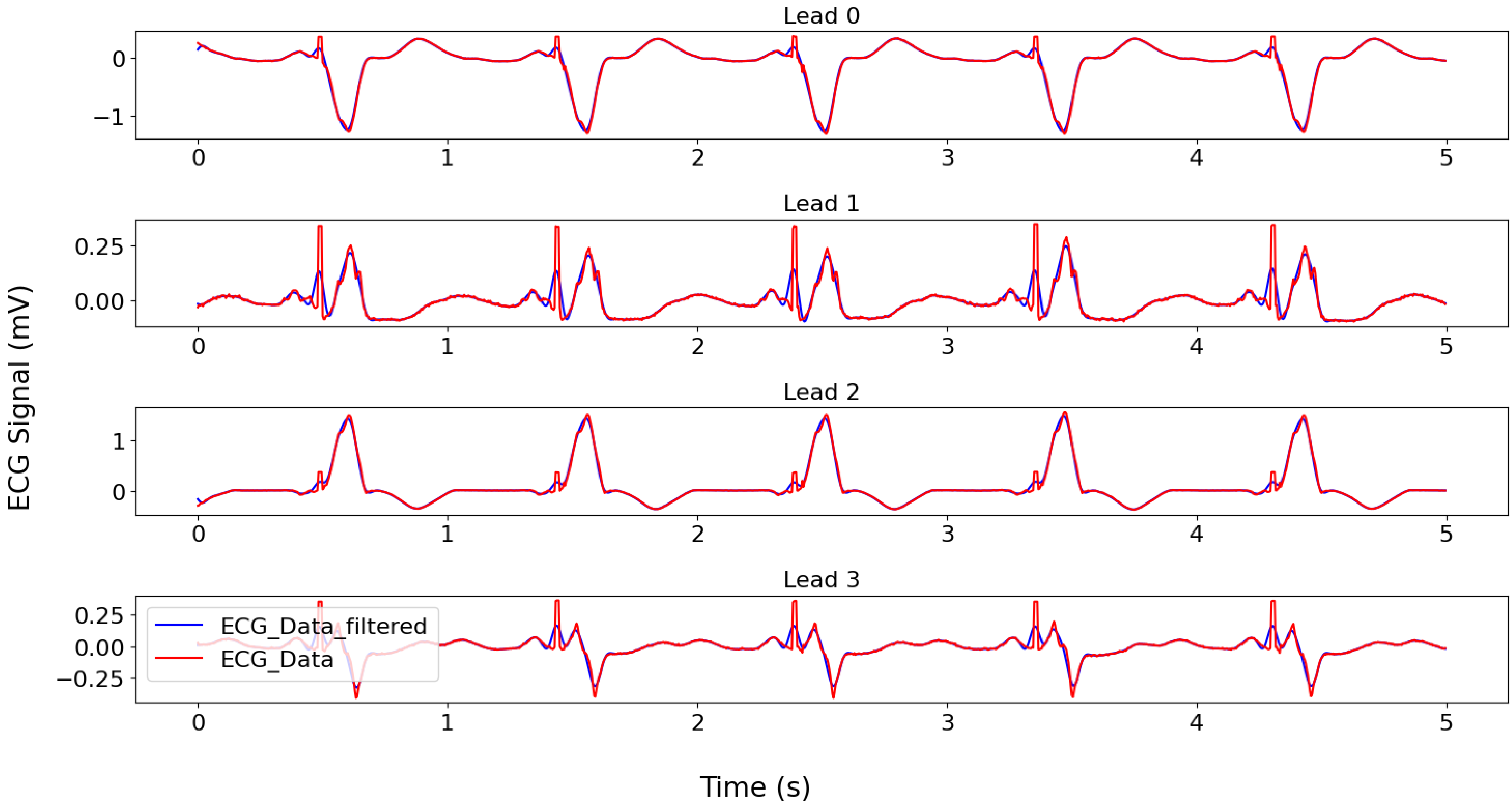

Supplement: Supplementary file 1 — OnlineSupplement_unmarked [file 44325_2025_75_MOESM1_ESM.pdf]
